# Environmental heterogeneity dominates the regulation of metabolites in ephemeral plants by the rhizosphere microbial community

**DOI:** 10.3389/fpls.2026.1815311

**Published:** 2026-07-27

**Authors:** Zhengwei Heng, Kangwei Jiang, Yu Wang, Chenkun Wang, Huiliang Liu, Lingwei Zhang

**Affiliations:** 1 Key Laboratory of Ecological Adaptation and Evolution of Extreme Environment in Xinjiang, College of Life Sciences, Xinjiang Agricultural University, Ürümqi, Xinjiang, China; 2 College of Grassland Sciences, Xinjiang Agricultural University, Ürümqi, Xinjiang, China; 3 State Key Laboratory of Ecological Safety and Sustainable Development in Arid Lands, Xinjiang Institute of Ecology and Geography, Chinese Academy of Sciences, Ürümqi, Xinjiang, China; 4 Xinjiang Key Lab of Conservation and Utilization of Plant Gene Resource, Xinjiang Institute of Ecology and Geography, Chinese Academy of Sciences, Ürümqi, Xinjiang, China; 5 Xinjiang Field Scientific Observation Research Station of Tianshan Wild Fruit Forest Ecosystem, Yili Botanical Garden, Xinjiang Institute of Ecology and Geography, Chinese Academy of Sciences, Xinyuan, Xinjiang, China

**Keywords:** co-occurrence network, ephemeral plants, heterogeneous habitat, metabolome, microbial community

## Abstract

High environmental heterogeneity in desert habitats likely drives the differentiation of plant metabolic regulation and rhizosphere microbial assembly; however, a systematic comparison of the coupling mechanisms between metabolites and microbial communities across diverse ephemeral plants and habitat gradients remains scarce. This study investigated four desert spring ephemeral species (Brassicaceae and Boraginaceae), comparing their whole-plant metabolites (integrating leaf and root metabolomes) metabolite profiles and rhizosphere microbial community structures across sandy, saline, and gravel desert habitats under naturally arid conditions. Results indicated that while metabolite compositions were generally similar across the four species, diversity patterns exhibited significant phylogenetic differentiation and habitat dependence. Specifically, metabolite alpha-diversity appeared to differ between the two families, whereas beta-diversity displayed species-specific habitat differentiation. Although rhizosphere microbial communities remained relatively conserved at the phylum level—suggesting an underlying environmental filtration effect—their diversity and network structures exhibited significant host-habitat interaction effects. Network complexity varied across habitats and decoupled from community stability, suggesting that environmental stress reshapes microbial assembly and interaction patterns. PLS-PM modeling further revealed that plant metabolite alpha-diversity is primarily linked to soil environmental factors indirectly through microbial diversity and network attributes, whereas no significant environmental-microbial regulatory pathway was observed for beta-diversity. Our study suggests the phylogenetic differentiation pattern of metabolic adaptation strategies in desert ephemeral plants against the background of taxonomic convergence, provides evidence for the mediating role of rhizosphere microbes in metabolic phenotypic regulation and their habitat dependence, and offers integrative insights for understanding plant-microbe co-adaptation mechanisms in desert ecosystems.

## 1 Introduction

Global aridification driven by climate change is accelerating, subjecting desert ecosystems to increasing ecological pressure (Gao et al., 2025). Currently, over 41% of the global land area is classified as arid or semi-arid, a proportion that continues to rise (Chen et al., 2025). It is important to note that “aridity” refers to a chronic, persistent climatic condition, whereas “drought” describes discrete extreme events that deviate from the normal water balance (Knapp et al., 2026). Against this backdrop, understanding how desert plants survive and reproduce in chronic water-limited arid environments has become a frontier issue in plant ecology. Ephemeral plants, a key functional group in Central Asian deserts, are characterized by short life cycles, synchronized growth rhythms, and high sensitivity to environmental fluctuations, making them an ideal model for studying adaptation mechanisms in extreme habitats (Zhang et al., 2026). However, existing research has largely focused on the physiological responses of individual species to single stressors (e.g., drought, salinity, or nutrient deficiency) (Lu et al., 2025). Systematic comparisons of metabolic strategies across different taxonomic lineages and their interactions with rhizosphere microbial communities across heterogeneous habitats remain limited. Desert ecosystems are not homogeneous but are complexes of diverse habitat types differing in water availability, salinity, soil texture, and nutrient levels, thereby forming spatially interwoven selection pressures (Liu et al., 2025). Different taxa may exhibit divergent metabolic strategies when facing the same stress due to their distinct secondary metabolic backgrounds (Pierce et al., 2021). Consequently, fully decoding the adaptation mechanisms of desert plants to naturally arid conditions requires a comparative framework that integrates both habitat types and taxonomic backgrounds (Palazon and Alcalde, 2025; Knapp et al., 2026).

Plant metabolic composition is not merely a passive response to stress but a functional manifestation of carbon and nitrogen allocation trade-offs under resource constraints (Xu and Gaquerel, 2025). In arid and nutrient-poor desert soils, limited carbon assimilation prompts resource reallocation among growth, defense, and osmotic regulation, reshaping metabolic networks. While primary metabolism maintains energy homeostasis, enhanced secondary metabolism reflects a shift toward defense and signaling (Palazon and Alcalde, 2025). These metabolic shifts further influence rhizosphere microbial assembly by altering root exudate composition and the chemical environment (Ma et al., 2025). Rhizosphere microbes, highly sensitive to host metabolism, contribute to plant stress resistance through nutrient transformation and signal modulation (Singh et al., 2025). Thus, metabolic regulation and microbial assembly represent a coupled dynamic process. This coupling is particularly crucial for ephemeral plants; their compressed life cycles intensify temporal selection pressures, requiring rapid metabolic remodeling and a potential reliance on microbial synergy for resource efficiency (Lu et al., 2025). Whether different ephemeral taxa drive convergent or divergent microbial assembly through differentiated metabolic adjustments remains to be elucidated.

The Gurbantünggüt Desert in Xinjiang is a representative temperate arid ecosystem. Controlled by a strong continental climate, the region experiences scarce precipitation and high evaporation, creating persistent water limitations (Zhang et al., 2026). Within this framework, three distinct habitats exist: sandy, saline, and gravelly deserts. These habitats vary significantly in edaphic factors and physical structure (Ding et al., 2025). Sandy deserts are dominated by mobile or semi-fixed dunes with coarse-grained soils and low water-holding capacity. Saline deserts feature high surface salt accumulation, creating a water-salt coupled stress distinct from mere aridity (Yin et al., 2026). Gravelly deserts are characterized by gravel cover, which regulates soil thermal environments and water redistribution. These habitats provide a natural gradient to explore plant and microbial responses across diverse environmental dimensions.

This study selected four dominant spring ephemeral species from the Brassicaceae (*Strigosella scorpioides* and *Meniocus linifolius*) and Boraginaceae (*Nonea caspica* and *Lappula myosotis*) families. These families represent independent evolutionary lineages with distinct metabolic backgrounds, providing an opportunity to explore potential phylogenetic influences on metabolic traits. We systematically analyzed their metabolomic profiles and rhizosphere microbial structures in this temperate arid ecosystem to address the following: (1) To what extent do metabolite compositions exhibit convergent or divergent patterns across heterogeneous environments? (2) How do rhizosphere microbial diversity and network structures respond to host-habitat interactions, and are they coupled with plant metabolic phenotypes? (3) Are there hierarchical association pathways between soil factors, microbial communities, and metabolites, and are these pathways habitat-dependent? Through cross-species and cross-habitat comparisons, we aim to reveal the coupling patterns between plant metabolism and rhizosphere microbial assembly under naturally arid conditions, and to deepen our ecological understanding of the adaptation strategies of ephemeral plants under environmental heterogeneity.

## 2 Materials and methods

### 2.1 Overview of the study area

The study area is situated in the southern Gurbantünggüt Desert of Xinjiang (44°11’ N - 46°20’ N, 84°31′E - 90°00’ E), positioned between the Tianshan and Altai Mountains and bordered by the western Junggar Mountains to the west. Its distinct geography and geomorphology have fostered diverse desert landscapes. The region experiences a typical temperate continental desert climate, characterized by hot, arid summers and prolonged, severe winters. The mean annual temperature (MAT) ranges from 6 to 10 °C, with a mean annual precipitation (MAP) of approximately 150 mm and an annual potential evaporation exceeding 2000 mm (Wang et al., 2011). Edaphic conditions are diverse, primarily featuring aeolian sandy, brown desert, and gray desert soils, with localized occurrences of takyric, meadow, and saline-alkali soils (Du et al., 2021). As the primary distribution center for ephemeral plants in China, this region supports a functional group highly sensitive to environmental fluctuations, including species such as *Strigosella scorpioides*, *Meniocus linifolius*, *Nonea caspica*, *Lappula myosotis*, and *Erodium oxyrhinchum*.

### 2.2 Experimental design

This study employed a spatially stratified sampling strategy (Stevens and Olsen, 2004), selecting adjacent sandy, gravelly, and saline desert areas within the Gurbantünggüt Desert as sampling plots. Following a representative sampling approach, six sites were established within each of the three ecological gradient zones, totaling 18 sampling sites separated by distances exceeding 10 km. At each site, a 10 m × 10 m macro-quadrat was established, within which five 2 m × 2 m micro-quadrats were positioned using a standard five-point sampling method. Quadrat selection adhered to three criteria: (1) wide and even distribution of the four target species; (2) provision of a 10–20 m marginal buffer zone; and (3) preference for flat or uniformly sloping terrain, avoiding transitional micro-topographies such as slope crests and valley bottoms (Fang et al., 2009). To minimize potential confounding effects of microhabitat heterogeneity, all quadrats were located in areas with consistent macro-topography, similar vegetation cover, and no large animal disturbance. Sampling was conducted on April 20, 2025, during the peak growth period of the ephemeral species. Plants of uniform size were carefully excavated to ensure root system integrity. Plants from five micro-quadrats were sampled separately according to species, and leaves and roots were separated. Four root samples and four leaf samples were obtained from each sampling point, resulting in a total of 144 plant samples from 18 sampling points. All samples were cleaned with deionized water, wrapped in aluminum foil, and the leaves and roots were used separately for subsequent metabolomics analysis (extracted and analyzed separately) to obtain independent metabolomics data for leaves and roots.

Concurrently, rhizosphere soil tightly adhering to the roots was collected and pooled by host species across the five micro-quadrats. Each pooled rhizosphere sample was halved; one portion (yielding 72 samples overall) was placed in sterile centrifuge tubes, snap-frozen in liquid nitrogen alongside the plant samples, and stored at −80 °C for subsequent metabolomic and microbial community analyses. The remaining portion (72 samples) was cleared of gravel and root debris, passed through a 2 mm sieve, and stored in sealed bags for air-drying and subsequent physicochemical characterization. Additionally, bulk soil from the top 20 cm was randomly collected using a soil auger across each macro-quadrat and homogenized to form a single background soil sample per site. This resulted in 18 non-rhizosphere soil samples, which were similarly processed for physicochemical analysis.

### 2.3 Sample determination

Metabolomics assays were performed independently on leaf and root samples. The specific steps are as follows: 100 mg of leaf (or root) tissue was accurately weighed into a 2 mL centrifuge tube containing a 6 mm diameter grinding bead and 400 µL of extraction buffer (methanol:water = 4:1, v/v). The extraction buffer was spiked with four internal standards: L-2-chlorophenylalanine, L-2-chlorophenylalanine-13C9, metoprolol, and sorbitol (each at 0.02 mg/mL). Samples were homogenized in a cryo-tissue homogenizer for 6 min (-10 °C, 50 Hz) and subjected to low-temperature ultrasonic extraction for 30 min (5 °C, 40 kHz). After standing at -20 °C for 30 min, the homogenates were centrifuged at 13, 000 × g for 15 min at 4 °C, and the supernatants were transferred to auto-sampler vials for analysis. LC-MS analysis was performed using an ultra-high performance liquid chromatography-tandem Fourier transform mass spectrometry (UHPLC-Q Exactive) system. Chromatographic separation was achieved on an ACQUITY UPLC BEH C18 column (100 mm × 2.1 mm i.d., 1.7 µm; Waters, Milford, MA, USA). The mobile phase consisted of 2% aqueous acetonitrile containing 0.1% formic acid (A) and pure acetonitrile containing 0.1% formic acid (B). The gradient elution program was: 0–1 min, 5% B; 1–9 min, 5%-95% B; 9–12 min, 95% B; 12-12.1 min, 95%-5% B; 12.1–15 min, 5% B. The flow rate was 0.35 mL/min, injection volume 3 µL, and column temperature 40 °C. Mass spectrometry data were acquired in both positive and negative electrospray ionization (ESI) modes, with a scan range of m/z 70-1050, Full MS resolution 70, 000, MS² resolution 17, 500, and stepped collision energies of 20, 40, and 60 eV. Sheath gas flow rate was 50 arb, auxiliary gas flow rate 13 arb, source temperature 450 °C, capillary temperature 320 °C; spray voltage was +3500 V in positive mode and -3000 V in negative mode. The injection volume was 3 µL, and the column temperature was maintained at 40 °C. Quality control (QC) samples were prepared by mixing equal volumes of all sample extracts. A QC sample was inserted after every 5–15 analytical samples to monitor instrument stability. Variables with a relative standard deviation (RSD) > 30% in the QC samples were discarded.

After LC-MS analysis, the raw data were imported into the metabolomics processing software Progenesis QI v3.0 (Waters Corporation, Milford, MA, USA) for baseline filtering, peak detection, integration, retention time correction, and peak alignment, generating a data matrix containing retention time, mass-to-charge ratio, and peak intensity information. Meanwhile, the MS and MS/MS spectral data were matched against the Majorbio in-house plant metabolite database (MJDBPM), with a mass error tolerance set to less than 10 ppm, and metabolites were identified based on MS/MS spectral matching scores. The data matrices obtained from both chromatographic columns were combined, deduplicated, and uploaded to the Majorbio Cloud Platform (cloud.majorbio.com) for further analysis. Prior to analysis, the data matrix was preprocessed as follows: missing values were filtered using the 80% rule, retaining variables with non-zero values in at least 80% of samples in at least one group; missing values were then imputed using the minimum value of the original matrix. To minimize technical variation arising from sample preparation and instrument instability, total sum normalization was applied to the peak intensity responses of the samples. Subsequently, variables with a relative standard deviation (RSD) > 30% in quality control (QC) samples were removed, and a log10 transformation was performed, yielding the final data matrix for subsequent analyses. The metabolomics data have been deposited to MetaboLights repository with the study identifier MTBLS14369.

Soil physicochemical properties were determined using standard methods: soil moisture content (SM) was determined using the oven-drying method; soil organic carbon (SOC) by potassium dichromate oxidation; total nitrogen (TN) by Kjeldahl digestion; total phosphorus (TP) by sodium hydroxide fusion followed by molybdenum-antimony colorimetry; ammonium nitrogen (NH_4_^+^-N) by alkaline diffusion; and available phosphorus (Av-P) by ammonium acetate extraction combined with flame photometry. Soil pH and electrical conductivity (EC) were measured in a 1:5 soil-to-water suspension using corresponding electrodes, following the protocols described by DeForest and Moorhead (2020). Detailed physicochemical profiles of the rhizosphere soils for the four species are provided in **Supplementary Figure S1**.

Total genomic DNA was extracted from soil samples using the E.Z.N.A.^®^ Soil DNA Kit (Omega Bio-Tek, Norcross, GA, USA) according to the manufacturer’s instructions. DNA quality was checked on 1% agarose gel, and DNA concentration and purity were determined using a NanoDrop2000 spectrophotometer (Thermo Scientific, USA). Using the mixed DNA as template, the V3-V4 hypervariable region of the bacterial 16S rRNA gene was amplified with barcoded primers 338F (5’-ACTCCTACGGGAGGCAGCAG-3’) and 806R (5’-GGACTACHVGGGTWTCTAAT-3’), and the fungal ITS1 region was amplified with primers ITS1F (5’-CTTGGTCATTTAGAGGAAGTAA-3’) and ITS2R (5’-GCTGCGTTCATCGATGC-3’). The PCR reaction mixture (20 μL) contained 4 μL of 5× TransStart FastPfu buffer, 2 μL of dNTPs (2.5 mM each), 0.8 μL of each primer (5 μM), 0.4 μL of TransStart FastPfu DNA polymerase, and 10 ng of template DNA. PCRs were performed on an ABI GeneAmp^®^ 9700 thermocycler using the following program: pre-denaturation at 95 °C for 3 min; followed by cycling: denaturation at 95 °C for 30 s, annealing at 55 °C for 30 s, extension at 72 °C for 30 s (27 cycles for bacteria, 32 cycles for fungi); final extension at 72 °C for 10 min; and held at 4 °C. PCR products were separated by 2% agarose gel electrophoresis, purified using a DNA Gel Extraction and Purification Kit (Yuhua, China), and quantified using a Qubit 4.0 fluorometer (Thermo Fisher Scientific, USA). Libraries were constructed using the NEXTFLEX^®^ Rapid DNA-Seq Kit, and paired-end sequencing was performed on the Illumina Nextseq2000 platform.

During sequence processing, fastp (v0.19.6) was used for quality control, and FLASH (v1.2.11) was used to merge paired-end sequences. The parameters were set to a minimum overlap of 10 bp and a maximum mismatch rate of 0.2. The quality control sliding window was 50 bp, and the average quality threshold was Q20 (Magoč and Salzberg, 2011). High-quality sequences were clustered into operational taxonomic units (OTUs) at 97% similarity using UPARSE v7.1 (Edgar, 2013). Chimeras were removed using UCHIME (gold.fa reference database), and singletons (OTUs with only one read) were discarded. To minimize the effects of sequencing depth on diversity analyses, all samples were rarefied to 20, 000 sequences. Taxonomic assignment of representative OTU sequences was performed using the RDP classifier v2.11 (Wang et al., 2007) with a 70% confidence threshold against the SILVA database (Release 138.1) for bacteria and the UNITE database (Release 10.0) for fungi. Absolute quantification: Spike-in OTUs were identified from the sequence dataset; a standard curve was generated for each sample based on the sequencing reads of the spike-in DNA (synthetic DNA with known copy numbers), and the absolute copy number of each OTU was calculated accordingly. The initial microbial sequencing data are hosted in the NCBI Sequence Read Archive database under accession numbers PRJNA1426877.

### 2.4 Data processing

All statistical analyses and data visualization were performed in R software (version 4.3.2), with the following packages: vegan 2.6-4, ggplot2 3.4.2, igraph 1.4.3, lme4 1.1-34, plspm 0.5.1, randomForest 4.7-1.1, emmeans 1.8.5, multcomp 1.4-23, ggClusterNet, among others. The vegan package (Oksanen et al., 2012) was utilized to calculate the Shannon index and Bray-Curtis dissimilarity, assessing the alpha diversity and beta-diversity of plant metabolites, respectively. It is worth noting that, to represent whole-plant metabolic profiles, metabolite data from leaves and roots of the same individual were combined (i.e., merged the peak tables of leaf and root metabolomes, retaining all metabolite features). Metabolite α-diversity, β-diversity, and subsequent differential metabolite analyses were performed based on this combined whole-plant metabolome dataset. This merging strategy ensures that whole-plant metabolite diversity comprehensively accounts for the metabolic contributions of both aboveground and belowground organs. Here, metabolite alpha diversity denotes the richness and evenness of metabolites within a single species, whereas beta diversity reflects the compositional dissimilarity among species. Shared metabolites across the three habitats were identified using Venn diagrams generated with the VennDiagram package (Chen and Boutros, 2011). Similarly, operational taxonomic unit (OTU) tables were employed to evaluate microbial diversity and community composition. Specifically, the Shannon index was calculated for all, abundant, and rare taxa to quantify alpha diversity, while Bray-Curtis dissimilarity was used for beta-diversity. Principal coordinate analysis (PCoA) based on Bray-Curtis distances was used to visualize the distribution patterns of each subcommunity across different habitats, and analysis of similarities (ANOSIM) was performed to statistically test the significance of these structural differences. To quantitatively disentangle the relative contributions of habitat heterogeneity and host plant identity to bacterial and fungal community structures, we performed variance partitioning analysis (VPA) using the vegan package. Prior to VPA, the species abundance matrix was Hellinger-transformed.

Co-occurrence networks for the rhizosphere microbial communities of the four plant species were constructed using the igraph package (Csardi and Nepusz, 2006), with bacterial and fungal datasets merged to build a bacteria-fungal co-occurrence network. Prior to constructing the network, microorganisms were screened based on OTUs with relative abundance below 0.01% and a frequency of fewer than 5 occurrences per treatment. Spearman correlation matrices were calculated, retaining only robust correlations (p < 0.05, |r| ≥ 0.7) as network edges. Network topological features—including node and edge counts, average degree, clustering coefficient, average path length, network diameter, and graph density—were extracted using the ggClusterNet package (Wen et al., 2022). Generally, higher values for node/edge counts, average degree, clustering coefficient, and graph density, coupled with shorter average path lengths and network diameters, indicate a denser, more complex network structure. To accurately reflect network complexity, the inverse transformations (X^-1^) of average path length and network diameter were applied. A network complexity index was then derived from the average of these seven standardized topological parameters. Finally, co-occurrence networks were visualized and modularized using Gephi (version 0.9.6). Following Xun et al. (2021), average variability (AVD) was calculated to comprehensively assess changes in species abundance; lower AVD values correspond to higher community stability.

Differences in rhizosphere microbial diversity, network complexity, stability, and SMF were analyzed via one-way ANOVA. To accurately evaluate specific group differences, *post hoc* multiple comparisons based on estimated marginal means (EMMs) were conducted using the emmeans package (Searle et al., 1980; Lenth and Piaskowski, 2026). Furthermore, significant differences were assigned and grouped using the multcomp package (Hothorn et al., 2008). Statistical significance was determined at *p* < 0.05. A random forest model using the randomForest package (Liaw and Wiener, 2002) evaluated the relative contributions of soil environmental factors to plant metabolite diversity, with parameters set as follows: ntree = 500, mtry = 1/3 of the number of variables (default), minimum node size = 5; variable importance was assessed using permutation importance. Relationships among microbial community diversity, network attributes, and plant metabolic diversity were assessed via general linear models using the lme4 package (Bates et al., 2015). Lastly, partial least squares path modeling (PLS-PM) was conducted using the plspm package (Sanchez, 2013), with models validated using goodness-of-fit indices.

Finally, partial least squares path modeling (PLS-PM) was performed using the plspm package in R (Sanchez, 2013). The multi-indicator latent variable was constructed in a reflective mode, whereas microbial diversity, network complexity, and network stability were incorporated as single-indicator constructs. The structural and measurement models were evaluated using the following quality indices: the goodness-of-fit (GoF > 0.5 indicates acceptable overall alignment); and the internal consistency and convergent validity of the blocks were assessed via Cronbach’s α (threshold > 0.7), composite reliability (CR, threshold > 0.7), and average variance extracted (AVE, ideally > 0.5, though values > 0.35 are acceptable in ecological models under high environmental heterogeneity).

## 3 Results

### 3.1 Habitat characteristics of annual ephemeral plants

**Figure 1:**
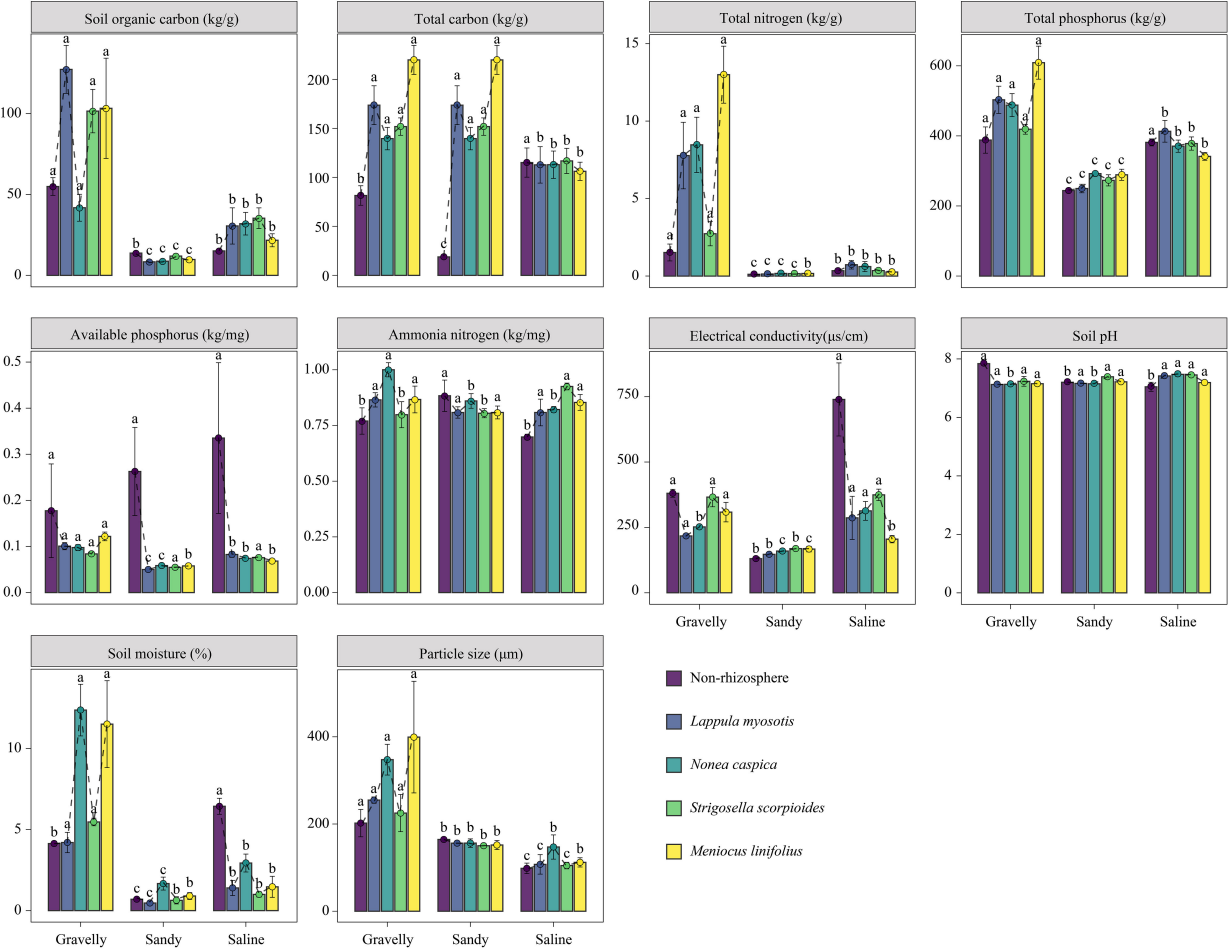
Rhizosphere soil physicochemical properties of *Lappula myosotis*, *Nonea caspica*, *Strigosella scorpioides*, and *Meniocus linifolius* in different habitats. Different lowercase letters indicate significant differences between different habitats (p < 0.05).

We compared three habitats—sand desert, saline desert, and gravelly desert—and found that there were significant differences in the soil physicochemical properties among the three habitats (**Figure 1**). Gravelly desert soils possessed significantly higher water, carbon, nitrogen, and phosphorus contents, whereas saline desert soils exhibited elevated electrical conductivity, and sandy desert soils displayed the poorest nutrient availability (ANOVA, *p* < 0.05). These findings confirm high environmental heterogeneity among the three habitats. Furthermore, compared with their respective bulk soils, the rhizosphere soils of the four ephemeral species generally exhibited higher values of organic carbon, total carbon, total phosphorus, and soil moisture. However, available phosphorus and total nitrogen did not consistently show rhizosphere enrichment in certain habitats (**Figure 1**), indicating that the accumulation patterns of different nutrient elements in the rhizosphere vary.

### 3.2 Changes in the composition and diversity of metabolites of four annual ephemeral plants in different habitats

**Figure 2:**
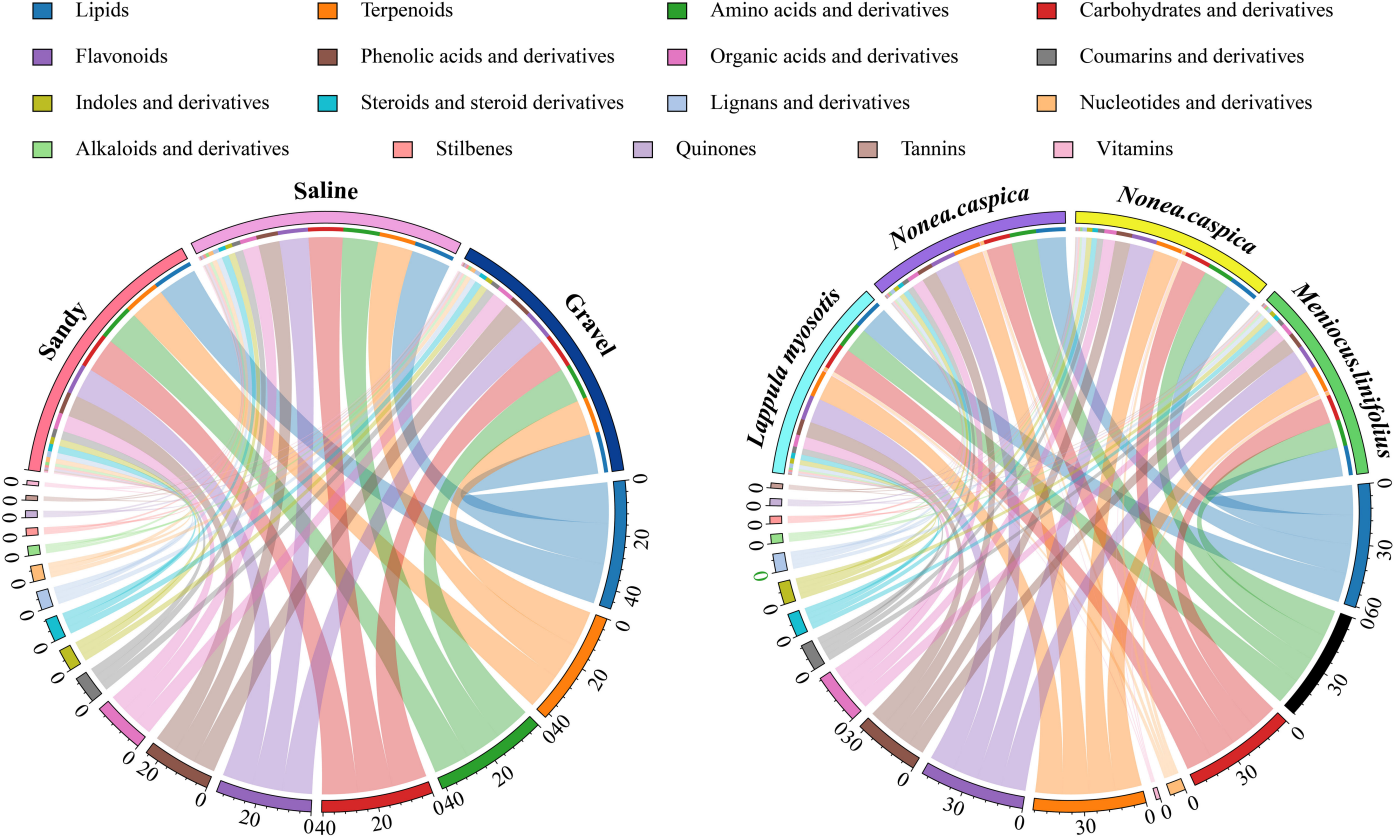
The composition of metabolites of *Lappula myosotis*, *Nonea caspica*, *Strigosella scorpioides* and *Meniocus linifolius* under different habitats.

Based on whole-plant metabolome data integrating leaf and root metabolites, metabolomic profiling of *Strigosella scorpioides*, *Meniocus linifolius*, *Nonea caspica*, and *Lappula myosotis* across the three habitats identified a broad spectrum of compounds, including lipids (15.56%), terpenes (13.92%), amino acids and their derivatives (13.81%), carbohydrates and their derivatives (13.51%), flavonoids (11.58%), phenolic acids and their derivatives (8.35%), organic acids and their derivatives (6.39%), coumarins and their derivatives (3.28%), steroids and their derivatives (2.79%), indoles and their derivatives (2.65%), lignans and their derivatives (2.24%), nucleotides and their derivatives (1.89%), alkaloids and their derivatives (1.16%), stilbenes (0.91%), quinones (0.84%), tannins (0.57%), and vitamins (0.53%). Lipids constituted the dominant primary metabolites across all samples, whereas terpenes were the most abundant secondary metabolites (**Figure 2**). Overall, the metabolite profiles of all samples shared a similar basic composition, with the proportions of different compound classes fluctuating across habitats. However, each of the four plant species possessed a set of unique metabolites in each habitat (**Supplementary Figure 1**). Analysis of these habitat-specific unique metabolites revealed that secondary metabolites, particularly flavonoids, constituted the majority, although the adaptive strategies and response intensities varied among species (**Supplementary Figure 2**).Further examination showed clear differentiation of unique metabolites depending on habitat, species, and family. In gravelly desert, flavonoid glycosides and anthocyanins were consistently enriched, with S. scorpioides (Brassicaceae) accumulating the highest number of flavonoid glycosides (e.g., isorhamnetin 3-(6’’-malonylglucoside)). In saline desert, stress-responsive metabolites such as anthocyanins (in *M. linifolius*) and shikonin (in *N. caspica*) were promoted. In sandy desert, basic flavonoids (e.g., luteolin) and cyanogenic glycosides dominated. Between the two families, the Brassicaceae species (*S. scorpioides* and *M. linifolius*) were enriched in isoflavones and O-methylated flavonoids, whereas the Boraginaceae species (*N. caspica* and *L. myosotis*) were enriched in luteolin, apigenin derivatives, stilbenes, and naphthoquinones. Detailed lists are provided in **Supplementary Table 2**.

**Figure 3:**
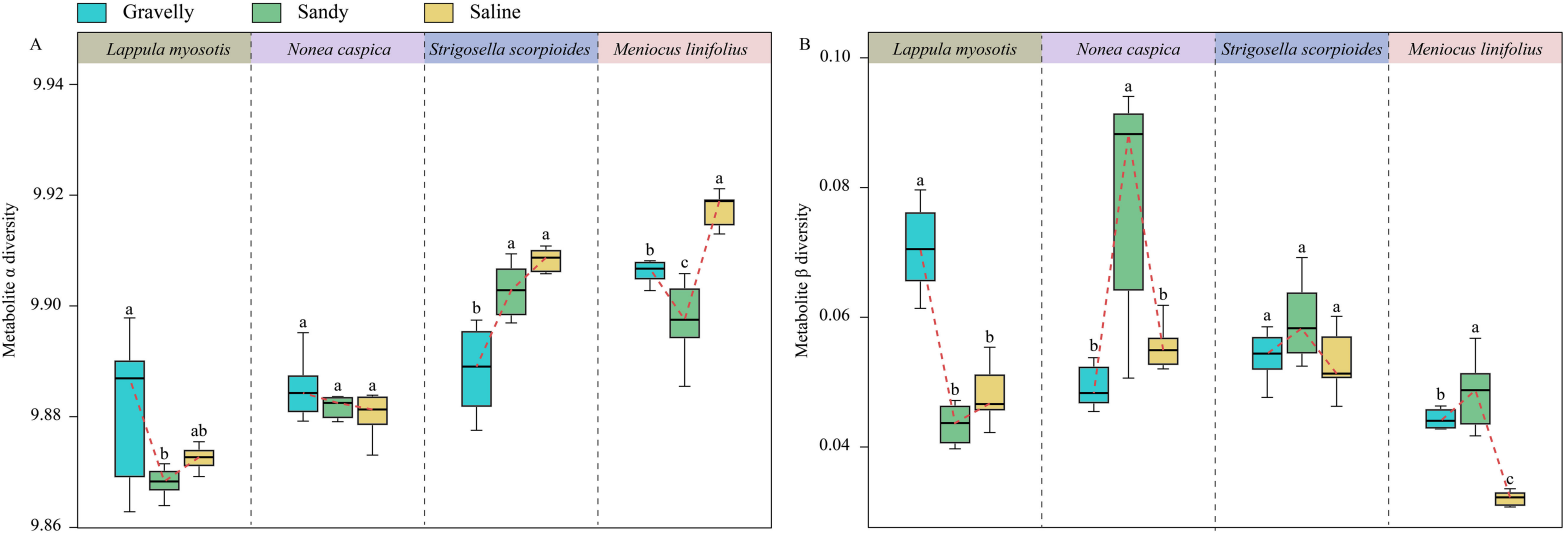
Metabolite diversity of annual ephemeral plants in sandy desert, saline desert and gravelly desert habitats. **(A)** Metabolite alpha-diversity of *L. myosotis*, *N. caspica*, *S. scorpioides*, and *M. linifolius* in sandy, gravel, and saline deserts. **(B)** Metabolite beta-diversity of *L. myosotis*, *N. caspica*, *S. scorpioides*, and *M. linifolius* in sandy, gravel, and saline deserts. Different lowercase letters indicate significant differences between different habitats (*p* < 0.05).

Regarding metabolite diversity, the alpha-diversity in *L. myosotis* and *N. caspica* exhibited no significant variation across the three habitats. However, *S. scorpioides* displayed lower alpha-diversity in the gravelly desert compared to the sandy and saline deserts (ANOVA: *p* < 0.05), while *M. linifolius* reached its highest alpha-diversity in the saline desert (ANOVA: *p* < 0.05, **Figure 3**). For beta-diversity, L. myosotis exhibited higher values in the gravelly desert than in the other two habitats (ANOVA: *p* < 0.05), a pattern similarly observed for *N. caspica* in the sandy desert (ANOVA: *p* < 0.05). The metabolite beta-diversity of *S. scorpioides* showed no significant differences across habitats, whereas *M. linifolius* displayed the lowest beta-diversity in the saline desert (p < 0.05). Generally, the metabolite alpha-diversity of these annual ephemeral plants was lower in gravelly deserts, while beta-diversity was minimized in saline deserts.

### 3.3 Rhizosphere microbial communities of annual ephemeral plants in different habitats

Environmental heterogeneity drove distinct, habitat-specific distributions of bacterial and fungal communities. Regardless of these distributional shifts, the extreme conditions of desert ecosystems-such as oligotrophic soils, water scarcity, and saline-alkali stress-render environmental filtering a dominant process in microbial community assembly, allowing only a subset of highly adapted, stress-tolerant phyla to successfully colonize and maintain high relative abundances across habitats (Xu et al., 2020.). Consequently, the overall community composition across the three habitats was characterized by a highly conserved core set of phyla. At the phylum level, bacterial communities were dominated by Pseudomonadota, Actinobacteriota, Bacteroidetes, Chloroflexi, and Acidobacteriota, collectively accounting for over 75% of the total relative abundance across all habitats (**Supplementary Figure 3A**). Fungal communities were overwhelmingly dominated by Ascomycota and Basidiomycota, which together comprised over 70% of the total abundance across habitats (**Supplementary Figure 3B**).

Furthermore, the rhizosphere bacterial alpha-diversity exhibited distinct, species-specific responses to habitats (**Supplementary Figure 3C**). Specifically, for the Boraginaceae species (*N. caspica* and *L. myosotis*), bacterial alpha-diversity reached its maximum in the gravelly desert (*p* < 0.05). In contrast, for the Brassicaceae species, the diversity of *M. linifolius* was significantly lower in the saline desert than in the other two habitats, whereas *S. scorpioides* showed peak diversity in the saline desert (EMMs pairwise comparison: *p* < 0.05). For fungal communities, the rhizosphere alpha-diversity across all four plant species was significantly higher in both gravelly and saline deserts compared to sandy deserts (EMMs pairwise comparison: *p* < 0.05, **Supplementary Figure 3D**). PCoA confirmed the significant segregation of rhizosphere microbial community structures across different environments for all four plant species (ANOSIM, *p* < 0.01). Notably, bacterial communities exhibited more pronounced habitat-driven shifts (ANOSIM: *R* = 0.695 to 0.831, *p* = 0.001; **Supplementary Figures S4A–D**) compared to fungal communities (ANOSIM: *R* = 0.458 to 0.587, *p* ≤ 0.002; **Supplementary Figures S4E–H**). To further quantify the drivers of these structural shifts in both bacterial and fungal communities, VPA was applied (**Supplementary Figure 5**). For bacterial communities, environmental factors independently explained 49.9% of the variation, while host identity independently accounted for only 5.1% (**Supplementary Figure 5A**). Similarly, for fungal communities, environmental factors independently explained 51.7% of the variation compared to 10.9% by host identity (**Supplementary Figure 5B**). These disproportionate variance explanations explicitly demonstrate that the assembly of both microbial communities across these habitats is primarily driven by strong environmental filtering rather than host selection.

**Figure 4:**
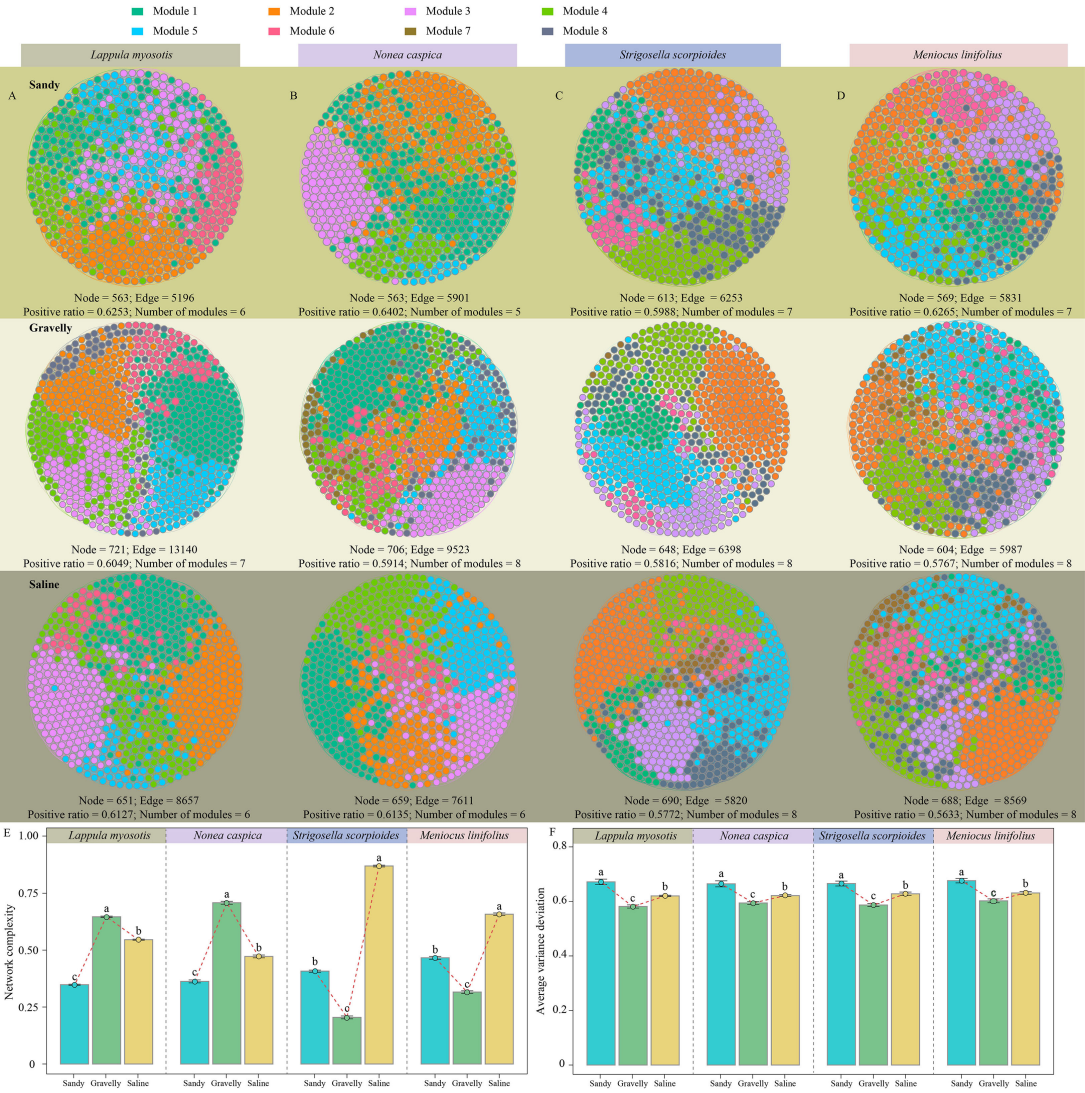
Co-occurrence network of rhizosphere microbial communities of annual ephemeral plants in sandy desert, gravelly desert, and saline desert habitats. **(A)** Co-occurrence network structure of rhizosphere soil microbial communities associated with *L. myosotis* in sandy, gravel, and saline deserts. **(B)** Co-occurrence network structure of rhizosphere soil microbial communities associated with *N. caspica* in sandy, gravel, and saline deserts. **(C)** Co-occurrence network structure of rhizosphere soil microbial communities associated with *S. scorpioides* in sandy, gravel, and saline deserts. **(D)** Co-occurrence network structure of rhizosphere soil microbial communities associated with *M. linifolius* in sandy, gravel, and saline deserts. **(E)** Complexity of the rhizosphere soil microbial co-occurrence networks associated with *L. myosotis*, *N. caspica*, *S. scorpioides*, and *M. linifolius* in sandy, gravel, and saline deserts, derived from the averaged normalized network topological properties. **(F)** Stability of the rhizosphere soil microbial communities associated with *L. myosotis*, *N. caspica*, *S. scorpioides*, and *M. linifolius* in sandy, gravel, and saline deserts, derived from the Average Variability of Species Abundance (AVD); a high AVD indicates low stability, and vice versa. Different lowercase letters indicate significant differences between different habitats (p < 0.05).

Rhizosphere microbial co-occurrence networks were predominantly driven by bacterial taxa across all four species and habitats (**Supplementary Figure 6**). The Boraginaceae species (*N. caspica* and *L. myosotis*) exhibited the most compact network structures and the highest number of modules in the gravelly desert, yet they possessed the lowest proportion of positive interspecific correlations (**Figures 4A, B**). In contrast, the Brassicaceae species (*S. scorpioides* and *M. linifolius*) displayed their most compact co-occurrence networks in the saline desert (**Figures 4C, D**). Notably, with the exception of network diameter and average path length, the topological metrics of the Boraginaceae rhizosphere networks were highest in the gravelly desert (**Supplementary Figures S7A, B**), whereas those for the Brassicaceae species peaked in the saline desert (**Supplementary Figures S7C, D**).

A comprehensive analysis of topological attributes demonstrated that variations in overall network complexity closely mirrored individual topological metrics. For the Boraginaceae species, network complexity was highest in the gravelly desert, being 85.6% and 95.0% higher than in the sandy desert, and 18.3% and 50.1% higher than in the saline desert (ANOVA: p < 0.05, **Figure 4E**). For the Brassicaceae species, network complexity was highest in the saline desert, being 113.0% and 41.0% higher than in the sandy desert, and 324.4% and 108.2% higher than in the gravelly desert, respectively (ANOVA: p < 0.05, **Figure 4E**). Assessing community stability via the AVD of microbial abundances revealed that the AVD was significantly lower in the gravelly desert than in other habitats. Since AVD is inversely proportional to community stability (Xun et al., 2021), these results indicate that rhizosphere microbial stability was significantly higher in the gravelly desert. (**Figures 4F**, ANOVA: *p* < 0.05).

### 3.4 Relationship between rhizosphere microbial community and metabolite diversity in different habitats of annual ephemeral plants

Linear regression analysis revealed that the alpha-diversity of plant metabolites was significantly and positively correlated with soil bacterial diversity across all three habitats (**Supplementary Figure 8A**). Specifically, the increase in plant metabolite richness showed high synchrony with the enhancement of bacterial community diversity. Among the four species, the correlation between metabolite alpha-diversity and bacterial diversity for *L. myosotis* was most pronounced in the gravelly habitat (linear regression: *R*^2^ = 0.79, *p* < 0.05). *N. caspica* and *S. scorpioides* exhibited the strongest goodness-of-fit in the sandy habitat (linear regression: *N. caspica*, *R*^2^ = 0.79, *p* < 0.05; *S. scorpioides*, *R*^2^ = 0.90, *p* < 0.01), while *M. linifolius* showed the best fitting effect in the saline habitat (*R*^2^ = 0.72, *p* < 0.05). Similar trends were observed for fungal communities (**Supplementary Figure 8B**), where *L. myosotis* demonstrated an exceptionally high correlation in the gravelly habitat (*R*^2^ = 0.88, *p* < 0.05), and *N. caspica* and *S. scorpioides* showed the strongest associations in the saline habitat (*R*^2^ = 0.79 and 0.81, respectively; *p* < 0.05).

In contrast, no significant correlations were found between plant metabolite beta-diversity (reflecting community structural differences) and soil microbial (both bacterial and fungal) diversity (**Supplementary Figures S8C, D**; *p* > 0.05). These results indicate that while soil microbial diversity significantly influences the overall richness/quantity of plant metabolites at the α level, it has limited explanatory power for the variation in metabolite compositional structure at the β level.

**Figure 5:**
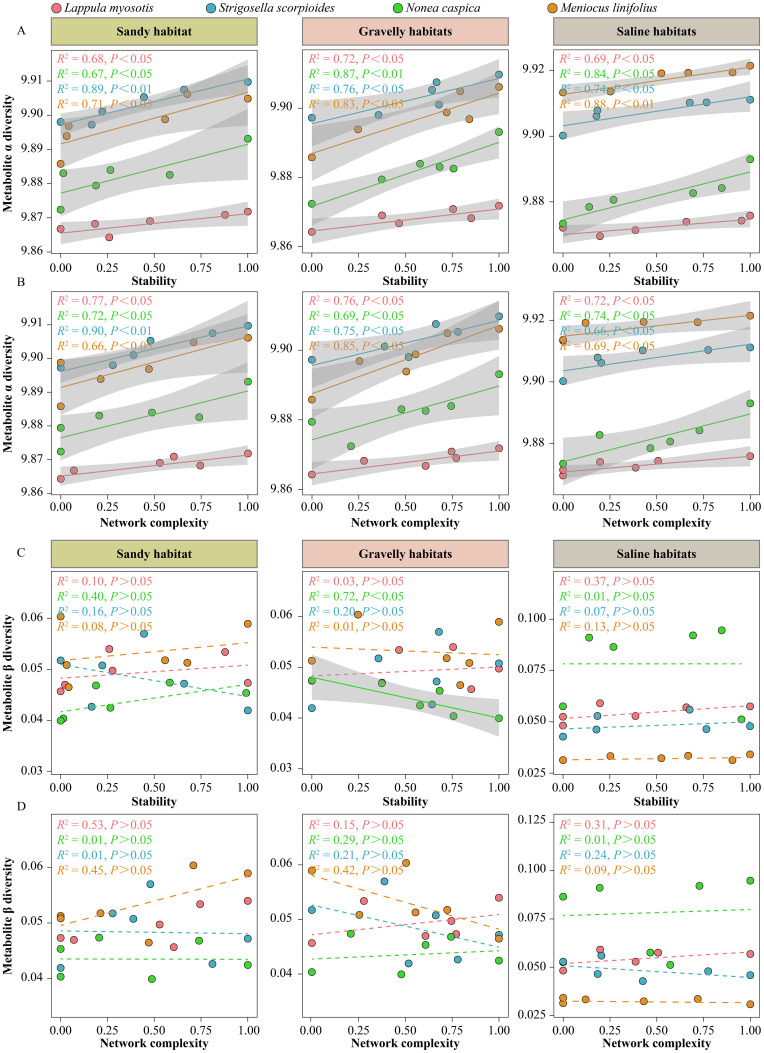
Correlation between rhizosphere microbial community co-occurrence networks and plant metabolites in sandy desert, gravelly desert, and saline desert habitats of four ephemeral plants. **(A)** Correlation between the stability of rhizosphere soil microbial communities and the α-diversity of plant metabolites in sandy desert, gravelly desert, and saline desert habitats. **(B)** Correlation between the network complexity of rhizosphere soil microbial communities and the α-diversity of plant metabolites in sandy desert, gravelly desert, and saline desert habitats. **(C)** Correlation between the stability of rhizosphere soil microbial communities and the β-diversity of plant metabolites in sandy desert, gravelly desert, and saline desert habitats. **(D)** Correlation between the network complexity of rhizosphere soil microbial communities and the β-diversity of plant metabolites in sandy desert, gravelly desert, and saline desert habitats.

Linear regression analysis revealed that rhizosphere microbial community stability was significantly and positively correlated with metabolite alpha-diversity (**Figure 5A**). Specifically, the correlations for *L. myosotis* and *N. caspica* were most pronounced in the gravelly habitat (linear regression: *R*^2^ = 0.72 and 0.87, respectively; *p* < 0.05 and *p* < 0.01). *S. scorpioides* exhibited the strongest goodness-of-fit in the sandy habitat (*R*^2^ = 0.89, *p* < 0.01), while *M. linifolius* showed the best fitting effect in the saline habitat (*R*^2^ = 0.88, *p* < 0.01). Similarly, microbial network complexity was also significantly and positively associated with metabolite alpha-diversity (**Figure 5B**). For *L. myosotis* and *S. scorpioides*, the tightest associations occurred in the sandy habitat (*R*^2^ = 0.72 and 0.87, respectively; *p* < 0.05 and *p* < 0.01). *N. caspica* displayed the highest goodness-of-fit in the saline habitat (*R*^2^ = 0.74, *p* < 0.05), whereas *M. linifolius* correlated most strongly in the gravelly habitat (*R*^2^ = 0.85, p < 0.05). Notably, consistent with the trends observed for microbial diversity, no significant correlations were found between microbial stability/complexity and metabolite beta-diversity (**Figures 5C, D**, *p* > 0.05). These findings suggest that stable and complex microbial systems primarily influence the overall richness of plant metabolites rather than their compositional dissimilarity.

**Figure 6:**
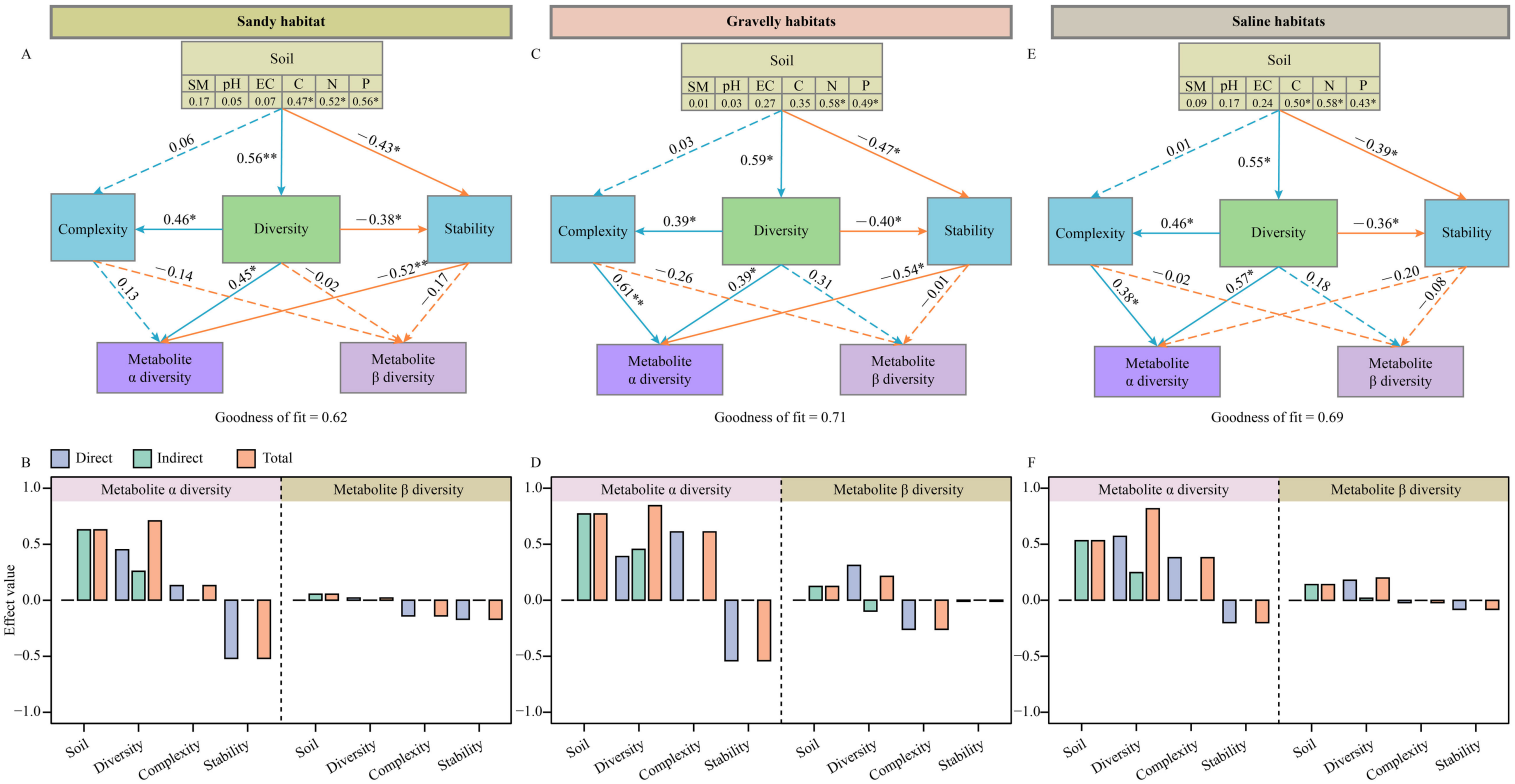
Partial least squares path modeling (PLS-PM) illustrating the effects of soil factors and microbial community properties on plant metabolite diversity across different habitats. **(A, C, E)** Directed path diagrams for the sandy habitat **(A)**, gravelly habitat **(C)**, and saline habitat **(E)**. Values within the “Soil” sub-table represent the outer loadings/weights of individual soil physicochemical indicators (SM, pH, EC, C, N, P) contributing to the “Soil” composite latent variable. Numbers adjacent to the arrows indicate standardized path coefficients (*β*). Blue and orange lines represent positive and negative effects, respectively. Solid and dashed arrows denote statistically significant (p < 0.05) and non-significant (p<0.05) pathways, respectively. Significance levels are indicated by *p < 0.05 and **p < 0.01. The goodness-of-fit (GoF) value is provided beneath each structural model. **(B, D, F)** Bar plots showing the direct, indirect, and total effects of predictors (Soil, microbial diversity, network complexity, and network stability) on plant metabolite alpha-diversity and beta-diversity in the sandy habitat **(B)**, gravelly habitat **(D)**, and saline habitat **(F)**. The blue line represents positive impact, and the red line represents negative impact. The number next to the arrow indicates the standardized path coefficient, the solid arrow indicates a significant effect, the dotted arrow indicates no significant effect, *: indicates a difference at the 0.05 level; **: indicates a significant difference at the 0.01 level.

To characterize the overall diversity of the microbial community, the Shannon indices of rhizosphere bacterial and fungal communities were standardized and averaged. A PLS-PM framework was then constructed to integrate soil properties, microbial network complexity, network stability, and plant metabolite alpha- and beta-diversity. All habitat-specific models yielded robust goodness-of-fit (GoF) values ranging from 0.62 to 0.71, validating the structural conceptualization of the models (**Figures 6A–C**); moreover, the reliability and validity metrics of the measurement models strictly met statistical requirements (**Supplementary Table 1**). The PLS-PM outputs revealed that the rhizosphere microbial community acted as the most critical regulator of plant metabolite alpha-diversity, although the driving pathways exhibited clear variations across habitats. In the sandy habitat, soil properties indirectly regulated metabolite alpha-diversity primarily by modulating microbial diversity (*β* = 0.56, p < 0.01) and network stability (*β* = -0.43, p < 0.05; **Figure 6A**); total effect analysis further confirmed that microbial diversity exerted the highest total deterministic contribution to metabolite alpha-diversity (**Figure 6B**). In the gravelly habitat, soil properties mediated changes in metabolite alpha-diversity via microbial diversity (*β* = 0.59, p < 0.05) and stability (*β* = -0.47, p < 0.05; **Figure 6C**); notably, while microbial diversity maintained the highest total effect, network complexity exhibited a stronger direct positive promotion on metabolite alpha-diversity (*β* = 0.61, p < 0.05; **Figure 6D**). In the saline habitat, soil properties similarly regulated metabolite alpha-diversity indirectly through microbial diversity (*β* = 0.55, p < 0.05) and network structure (**Figure 6E**), with the total effect of microbial diversity predominantly ruling the system (**Figure 6F**). Collectively, our modeling demonstrated that rhizosphere microbial attributes, coupled with soil physicochemical factors, jointly explained the variance in plant metabolite alpha-diversity. In contrast, neither soil properties nor any microbial community metrics exerted a significant regulatory effect on plant metabolite beta-diversity across all three desert habitats (p > 0.05, **Figures 6A–C**).

## 4 Discussion

### 4.1 Changes in metabolites of annual ephemeral plants in different habitats

Comparative metabolomic analysis of four ephemeral species from the Boraginaceae (*Nonea caspica and Lappula myosotis*) and Brassicaceae (*Strigosella scorpioides and Meniocus linifolius*) families elucidated their distinct metabolic adaptation strategies across heterogeneous desert habitats (sandy, saline, and gravelly) in Xinjiang, China. Although these species belong to divergent lineages and face varied abiotic constraints-including differences in water and salt availability, soil texture, and stress regimes-all samples exhibited a highly conserved baseline profile, dominated by lipids as the primary metabolites and terpenes as the major secondary metabolites. This convergence suggests that convergent core metabolic configurations may have evolved across distinct ephemeral lineages in response to chronic aridity, high irradiance, and extreme temperature fluctuations typical of Xinjiang deserts (Weng, 2014). Notably, this phenomenon is not unique to Xinjiang—desert plants from the Sonoran and Chihuahuan Deserts of North America similarly utilize terpenoids and phenolics as major surface natural products, forming a chemical barrier on their epidermal surfaces to prevent excessive cuticular water loss and protect against UV radiation damage; cross-continental comparisons indicate a high degree of functional conservation in defensive secondary metabolism among desert plants (Rodriguez, 1983). Lipids, crucial for maintaining cell membrane homeostasis and energy storage, play a dominant role, reflecting the universal physiological requirements of desert plants to cope with water deficits and thermal stress (Sharma et al., 2023). Concurrently, the ubiquitous enrichment of terpenoids is closely linked to their multifaceted roles in antioxidation, photoprotection, and herbivore deterrence, providing a broad-spectrum defense foundation against complex environmental stressors (Tholl, 2015). While Brassicaceae and Boraginaceae in non-arid environments typically exhibit strong, lineage-specific metabolomic divergence driven by local heterogeneity (Züst and Agrawal, 2017; Varela Alonso et al., 2022), the convergence observed here highlights how chronic aridity imposes severe uniform selection pressures. This forces phylogenetically disparate lineages to adopt a similar core defensive architecture.”

Despite sharing a conserved core metabolome, the relative abundances of specific compound classes fluctuated across habitats. Crucially, each species synthesized habitat-specific unique metabolites (**Supplementary Figure 1**), indicating that within a conserved framework, individual taxa retain the plasticity to mount localized chemical responses. Further analysis revealed that these unique metabolites were predominantly secondary compounds, particularly flavonoids (**Supplementary Figure 2**). This suggests potential ecological adaptation strategies. As pivotal products of the phenylpropanoid pathway, flavonoids mediate plant responses to UV-B radiation, oxidative stress, pathogen ingress, and herbivory (Yu et al., 2025; Bulut et al., 2025; Falcone Ferreyra et al., 2012). Given that desert environments are characterized by intense insolation, drought-induced oxidative stress, and fluctuating biotic pressures, the repeated enrichment of flavonoids as major habitat-specific metabolites implies they may be key chemical tools for local adaptation in ephemeral plants (Yang et al., 2025). Desert plants of the Arabian Peninsula exhibit similar metabolic strategies when responding to combined drought and heat stress, with significant activation of phenolic and flavonoid metabolic pathways to mitigate oxidative damage (Alhaithloul, 2019). The predominance of flavonoids in these unique metabolite pools further suggests that disparate plant lineages may convergently rely on the phenylpropanoid pathway as a core adaptive node when encountering analogous selective pressures. Unlike in non-arid systems where flavonoid accumulation is often transiently induced by stress (e.g., in temperate Brassica napus; Neugart et al., 2018), our findings suggest that long-term aridity selection drives the evolution of flavonoids into constitutive basal stress-tolerance metabolites.Analyses of metabolite alpha-diversity indicated that the intra-specific metabolomic profiles of the Boraginaceae species (*N. caspica* and *L. myosotis*) remained largely consistent across the three habitats, signifying robust metabolic homeostasis and resilience to environmental perturbations. This conclusion is validated by our PLS-PM results in both gravelly and saline deserts. In contrast, the Brassicaceae species exhibited significant habitat-driven variability: *S. scorpioides* displayed markedly reduced alpha-diversity in the gravelly desert, whereas *M. linifolius* exhibited its highest alpha-diversity in the saline desert (**Figure 3**). The significant reduction in alpha-diversity for *S. scorpioides* in gravelly habitats likely stems from a dual mechanism. First, the relatively favorable edaphic resource conditions in gravelly deserts (**Figure 1**) may attenuate stress heterogeneity, prompting a convergence toward a basal, energy-efficient metabolic state due to relaxed selective pressures (Figueroa-Macías et al., 2021; Züst and Agrawal, 2017). Second, dominant abiotic filters specific to gravelly habitats, such as mechanical resistance from gravel cover and pronounced thermal fluctuations, may force the allocation of resources into highly specialized pathways, thereby restricting the broader metabolic expression spectrum (Du et al., 2025). Conversely, the elevated alpha-diversity of *M. linifolius* in the saline desert reflects the activation of multiple parallel pathways induced by salt stress. Saline environments concurrently impose osmotic stress, ion toxicity, and oxidative damage; because a single pathway is insufficient to counteract these compounded stressors, expanding the metabolic repertoire becomes an obligatory adaptive response (Khan, 2025). The reduced metabolic plasticity observed in the Boraginaceae species implies that their metabolic networks possess a broader responsive framework or superior homeostatic buffering capacity.

Assessments of metabolomic beta-diversity further revealed habitat-driven, population-level metabolic differentiation. *N. caspica* and *L. myosotis* displayed elevated beta-diversity in gravelly and sandy deserts, respectively, suggesting intra-population metabolic divergence in response to complex microtopography or extreme hydrological fluctuations. Conversely, *M. linifolius* exhibited the lowest beta-diversity in the saline desert, highlighting the strong homogenizing selection exerted by salt stress on metabolic phenotypes. *S. scorpioides* exhibited no significant differences in beta-diversity across any habitat, indicating a highly conserved population-level metabolic phenotype that resists heterogeneity-induced differentiation (**Figure 3**). These patterns demonstrate that species deploy fundamentally different strategies to navigate habitat heterogeneity: some exploit microhabitat niches via inter-individual metabolic divergence, others converge phenotypically under dominant stressors, and highly conserved species maintain homogeneous population profiles (Khan, 2025). Notably, these strategies appeared to differ between the two families in this study. Collectively, the Brassicaceae species exhibited high metabolic plasticity, undergoing extensive phenotypic remodeling in response to habitat shifts. Conversely, Boraginaceae species demonstrated greater metabolic robustness, maintaining relatively stable diversity patterns across environmental gradients. It should be noted that, with only two species per family, the observed differences between the two families cannot be strictly distinguished from species-specific effects (e.g., habitat preference, phenology, root architecture, or microhabitat occupancy). These patterns should therefore be considered preliminary and await validation with a larger number of species per family.

### 4.2 Changes in the rhizosphere microbial community of annual ephemeral plants under different habitats

Phylum-level analysis revealed that despite significant variations in soil physicochemical properties and stress regimes among the three desert habitats, rhizosphere microbial communities maintained a highly conserved core taxonomic configuration at higher ranks (**Supplementary Figures S3A, B**). The widespread dominance of Actinobacteriota and Pseudomonadota in arid, saline, and oligotrophic environments is closely linked to their sporulation capabilities, potential to synthesize stress-resistant metabolites, and proficiency in degrading recalcitrant substrates (Yuan et al., 2021). The absolute dominance of Ascomycota in desert fungal communities aligns with their life-history traits, including high desiccation and salt tolerance, as well as robust saprophytic adaptability (Gong et al., 2023). Furthermore, principal coordinate analysis (PCoA) revealed significant habitat-specific segregation of both bacterial and fungal communities, with bacterial assemblages exhibiting more pronounced spatial partitioning than fungal communities (**Supplementary Figure 4**). This suggests that rhizosphere microbial assembly is co-driven by host-specific selections and the universal environmental filtering of desert habitats (Ma et al., 2025). The higher sensitivity of bacterial communities to environmental gradients may be attributed to their shorter generation times, higher metabolic plasticity, and more limited dispersal capacities compared to fungi (Wang et al., 2026). This macro-pattern is highly consistent with the findings of Zhang et al. (2024), who studied rhizosphere microbiomes of three desert plant species in Xinjiang and similarly documented differential habitat-driven assembly patterns for bacterial and fungal communities.

The distribution patterns of rhizosphere bacterial alpha-diversity across the four species appeared to differ between the two families. Specifically, the bacterial alpha-diversity of the Boraginaceae species (*Nonea caspica* and *Lappula myosotis*) was significantly higher in the gravelly desert compared to the sandy and saline deserts, whereas Brassicaceae species (*Strigosella scorpioides* and *Meniocus linifolius*) exhibited their highest bacterial alpha-diversity in the saline desert (**Supplementary Figure 3C**). These divergent trends indicate that rhizosphere bacterial enrichment is not strictly determined by baseline habitat resources but is strongly modulated by host-habitat interactions (Fitzpatrick et al., 2018; Trivedi et al., 2020). Previous studies have highlighted that soil texture and porosity significantly influence the diffusion gradients of root exudates, thereby shaping microbial recruitment (Sasse et al., 2018; Vives-Peris et al., 2020). Accordingly, the relatively loose soil structure of the gravelly desert may facilitate exudate diffusion, potentially allowing Boraginaceae species to assemble more diverse bacterial consortia. Conversely, the elevated bacterial alpha-diversity of Brassicaceae species in the saline desert may be linked to the remodeling of their root exudative profiles. Previous studies have demonstrated that plants specifically alter their root exudation under salt stress to recruit diverse, stress-tolerant microbial consortia (Pan et al., 2022). Notably, independent studies have documented the distinct rhizosphere influence of Boraginaceae plants: Panchenko et al. (2022) found that Boraginaceae species actively stimulated hydrocarbon-oxidizing microorganisms in their rhizosphere in oil-contaminated soils, suggesting a unique rhizosphere-regulating capacity of this family.

In stark contrast to bacterial patterns, the rhizosphere fungal alpha-diversity for all four species was uniformly higher in both gravelly and saline deserts than in the sandy desert (**Supplementary Figure 3D**). Although fungi are generally considered more drought-tolerant than bacteria, the extreme aridity and poor water retention of the sandy desert exceed their physiological thresholds, directly constraining spore germination and hyphal extension (Maestre et al., 2015; Preece et al., 2019). Consequently, fungal diversity exhibits distinct peaks in the relatively nutrient- and moisture-rich gravelly desert, where microclimatic conditions are more conducive to mycelial proliferation. Crucially, this observation is explicitly corroborated by our variance partitioning analysis (VPA) (**Supplementary Figure 5**). The VPA quantified that environmental factors independently explained 51.7% of the variation in fungal community structure, substantially outweighing the 10.9% explained purely by host identity (**Supplementary Figure 5B**). A parallel environmental dominance was also observed in bacterial communities (49.9% vs. 5.1%; **Supplementary Figure 5A**). These disproportionate effect sizes provide robust empirical evidence that fungal assembly is primarily driven by severe environmental filtering, with host identity exerting only a secondary effect (Xu et al., 2025). Studies of Brassicaceae host-soil feedbacks in dryland regions of the United States have similarly shown that under water-limited conditions, the regulatory effect of host plants on bacterial communities is significantly attenuated, with environmental factors—particularly soil moisture—becoming the dominant driver of community structure, aligning closely with our VPA findings (Blakney et al., 2022). While host genetics can significantly shape the microbiome in non-stressed or temperate environments (Bulgarelli et al., 2012; Peiffer et al., 2013), our findings indicate that under severe aridity, environmental filtering decisively outweighs host signals. This may shifts assembly towards a deterministic process primarily driven by environmental constraints.

Network analysis was employed to elucidate the habitat dependence and host specificity of interspecific microbial interactions. Boraginaceae species displayed the highest network complexity coupled with the lowest proportion of positive correlations in the gravelly desert (**Figures 4A, B**). This dynamic likely stems from a nonlinear relationship between resource availability and competitive intensity. This interpretation is directly anchored by our PLS-PM results for the gravelly desert (**Figure 6B**). The model demonstrates that while enhanced edaphic conditions significantly promote microbial diversity (path coefficient = 0.59), both the soil resources and the resulting high diversity exert strong negative effects on community stability (path coefficients = -0.47 and -0.40, respectively). Ecologically, although superior edaphic conditions support higher microbial diversity, the available rhizosphere carbon pool and colonization niches may not expand proportionally. This discrepancy intensifies competition for limited microsites, driving the observed community instability and increasing the proportion of negative network edges (Lin et al., 2021). This pattern is consistent with observations in hyperarid deserts, where the rhizosheath-root system functions as an “edaphic mini-oasis”: an enriched microenvironment that harbors greater microbial biomass and diversity but simultaneously exhibits intense inter-taxon competition (Marasco et al., 2022). Furthermore, studies of desert microbial co-occurrence networks have shown that in resource-rich microenvironments, networks are often dominated by negative edges, reflecting niche partitioning and competitive exclusion rather than cooperative interactions (Maurice et al., 2024). Thus, heightened network complexity in Boraginaceae species under gravelly desert conditions reflects an increased frequency of competitive interactions rather than enhanced mutualistic reciprocity. In this study, Boraginaceae species in gravelly desert exhibited the highest network complexity but the lowest proportion of positive correlations, reflecting intensified competition under relatively resource-rich conditions. This differs from observations in other ecosystems where resource richness often increases the proportion of positive correlations and cooperative interactions: for instance, in a microbial cross-feeding mutualism, high resource availability promotes cooperative interactions, whereas resource limitation shifts the interaction toward competition (Hoek et al., 2016). Such habitat-dependent divergence in interspecific interaction patterns is rarely so clearly separated by host lineage in non-arid ecosystems, suggesting that extreme aridity amplifies the ecological costs and benefits of lineage-specific adaptive strategies.

Conversely, Brassicaceae species exhibited maximum network complexity in the saline desert (**Figures 4C, D**). While severe salt stress typically reduces microbial diversity, the bacterial alpha-diversity associated with these two Brassicaceae species was significantly elevated in the saline habitat (**Supplementary Figure 3C**). This suggests that specific root exudate remodeling under salt stress actively filters and sustains diverse, salt-tolerant bacterial consortia (Pan et al., 2022). This strategy has been documented in multiple plant species, where salt-stressed roots reshape their exudation profiles to attract stress-alleviating rhizobacteria (Kumar et al., 2023). Consequently, the increased network complexity likely reflects strengthened positive interactions, wherein surviving taxa form synergistic metabolic associations to collectively mitigate environmental stress. Ultimately, the phylogenetic divergence between Boraginaceae and Brassicaceae in network assembly stems from the fundamentally different resource and stress profiles of their preferred habitats: the former constructs highly competitive networks in resource-rich environments, whereas the latter forms highly cooperative networks in stress-dominated settings. This strategic coupling provides a novel perspective on the ecological adaptation and phylogenetic differentiation of desert plants.

Furthermore, the stability of rhizosphere microbial communities across all four species was significantly higher in the gravelly desert than in the sandy and saline deserts (**Figure 4F**). This pattern partially uncouples from the trends observed in network complexity. While overall microbial community stability was highest in the gravelly desert across all taxa, the trends in network complexity exhibited distinct phylogenetic differentiation, leading to divergent coupling relationships between complexity and stability. Specifically, for Boraginaceae species (*Nonea caspica* and *Lappula myosotis*), both network complexity and community stability peaked simultaneously in the gravelly desert. In stark contrast, Brassicaceae species (*Strigosella scorpioides* and *Meniocus linifolius*) displayed their maximum network complexity in the saline desert, yet their community stability was highest in the gravelly desert (indicating a spatial decoupling). This clear contrast further underscores that under the severe osmotic stress of the saline desert, an increase in microbial network complexity-as seen in the Brassicaceae-does not necessarily translate into enhanced community stability against environmental disturbances. Microbial community stability may be modulated by a combination of environmental variability, disturbance frequency, and internal network features such as biodiversity and keystone taxa functions (Xun et al., 2021). In this study, the relatively stable interannual fluctuations and higher soil nutrient and moisture availability in the gravelly desert provided a predictable resource base; as suggested by De Vries and Shade (2013), such stable resource provisioning buffers microbial communities against environmental perturbations, thereby promoting higher overall stability. Conversely, although the saline desert supported highly complex networks for Brassicaceae species, intermittent osmotic shocks and ion toxicity events likely kept these communities in a non-equilibrium state, driving high average variability and reduced stability. Network complexity reflects an instantaneous snapshot of interaction intensity, whereas community stability represents the capacity to resist disturbance and maintain functional integrity over time (Cornell et al., 2023). These properties are likely governed by distinct ecological mechanisms; complexity is heavily influenced by localized resource abundance and host regulation, whereas stability is fundamentally constrained by environmental variability and disturbance history (Yuan et al., 2021). While some studies in less extreme environments link reduced network complexity to declining stability (Zheng et al., 2024), our data indicate this relationship is highly context-dependent. Under extreme osmotic stress, Brassicaceae species foster dense interspecific associations that reflect a fragile, stress-induced state rather than robust resilience, providing evidence that structural complexity does not universally guarantee ecological stability.

### 4.3 Regulation of metabolites by rhizosphere microbial communities in different habitats of annual ephemeral plants

Linear regression models revealed that the metabolite alpha-diversity of all four species was significantly and positively correlated with both soil bacterial and fungal diversity across the sandy, gravelly, and saline desert habitats. In contrast, metabolite beta-diversity exhibited no significant correlation with soil microbial diversity. These results indicate a robust, cross-habitat, and cross-species coupling between the richness of soil microbial communities and the complexity of individual plant metabolic profiles; however, microbial diversity does not appear to drive metabolic differentiation at the plant population level. The positive correlation between metabolite alpha-diversity and soil microbial diversity is likely maintained through a bidirectional feedback loop. First, diverse rhizosphere microbiomes can activate multiple secondary metabolic pathways in host plants by secreting growth regulators, mediating nutrient mobilization and detoxification, and inducing systemic resistance, thereby directly expanding the host’s metabolic repertoire (Wang et al., 2024). Conversely, hosts with richer metabolic profiles release more highly diverse root exudates, which sequentially recruit and sustain a more diverse rhizosphere microbial community (Gong et al., 2023). The stability of this synergistic relationship across different habitats and taxa suggests that this coupling mechanism is a universal feature among desert ephemeral plants. Furthermore, the lack of correlation between metabolite beta-diversity and soil microbial diversity is consistent with ecological theory. Population-level metabolic differentiation is primarily governed by phenotypic plasticity—driven by microenvironmental heterogeneity (e.g., light, moisture, and nutrients)—and the spatial distribution of genetic and epigenetic variations, rather than by the sheer capacity of the community-scale microbial species pool (Kim et al., 2022; Hayashi et al., 2023). Unlike nutrient-sufficient environments where plant-microbiome metabolic coupling can be weak or inconsistent (Züst and Agrawal, 2017), the robust correlations observed here suggest that extreme aridity converts this dynamic into a constitutive baseline strategy.

Additionally, the stability and network complexity of the rhizosphere microbial communities were significantly and positively correlated with plant metabolite alpha-diversity (**Figures 5A, C**). Notably, community stability in this study was quantified using the average variability of microbial abundances; thus, a lower value denotes higher stability. Consequently, higher plant metabolite alpha-diversity was associated with more volatile (higher average variability) and topologically complex microbial networks. This pattern implies that plant metabolic profiles become increasingly diversified in highly dynamic rhizosphere microenvironments. Conversely, as microbial communities stabilize, plant metabolite alpha-diversity decreases. The positive correlation between network complexity and metabolite alpha-diversity indicates that dense interspecific microbial interactions often coincide with varied metabolic profiles. In habitats characterized by fluctuating microbial communities and complex interaction networks, plants must activate a broader array of secondary metabolic pathways to navigate unpredictable biotic and abiotic stresses, resulting in enriched metabolic profiles (Anzano et al., 2022). In contrast, in habitats harboring stable and minimally disturbed microbial communities, plants may reduce their metabolic investment in intrinsic antimicrobial defense systems. This dynamic aligns with the classic growth-defense tradeoff hypothesis, postulating that in predictable and low-stress environments, plants prioritize resource allocation toward growth rather than diversifying their chemical defenses (Huot et al., 2014). As suggested by He et al. (2022), the stability of soil microbial community assembly significantly influences plant metabolic profiling, where the alleviation of biotic stress allows for a strategic reallocation of energy. Furthermore, the establishment of a stable rhizosphere microbiome can effectively optimize plant resource partitioning (Berendsen et al., 2012), potentially driving the observed convergence in plant metabolic expression within these more hospitable desert habitats. In contrast to mesic ecosystems where plant metabolism is primarily shaped by genetic and developmental factors rather than microbial feedback (Züst and Agrawal, 2017), the extreme aridity in our study system appears to intensify the reliance of plants on dynamic microbial interactions to expand their chemical defense repertoire.

Using partial least squares path modeling (PLS-PM), we further elucidated the hierarchical regulatory networks linking edaphic factors, microbial communities, and plant metabolite alpha-diversity (**Figure 6**). Across all three habitats, edaphic factors exerted no significant direct regulatory effect on plant metabolite alpha-diversity; instead, their influence was strictly indirect, mediated entirely by the microbial community. This establishes the rhizosphere microbiome as the central hub bridging the soil environment with the host plant’s metabolic phenotype (Wang et al., 2024; Ma et al., 2025). In essence, edaphic signals must be transduced into activation cues for the host’s secondary metabolic pathways via the maintenance of microbial diversity, the establishment of community stability, and the organization of interaction networks (Bi et al., 2021). Microbial community diversity exhibited the highest overall driving effect across all habitats, confirming its universal role as a core mediating variable—a finding that cross-validates the positive correlations observed in the linear regression models(**Figures 6B, D, F**). Furthermore, the habitat-specific differentiation of these regulatory pathways highlights the context-dependent importance of distinct microbial functional attributes. In sandy deserts (**Figure 6A**), where extreme water and nutrient scarcity imposes severe survival bottlenecks on the microbes themselves, community diversity (β = 0.56) and stability (β = –0.43) serve as the primary mediators of edaphic signals (Leung et al., 2020). In gravelly deserts (**Figure 6C**), characterized by relatively favorable edaphic conditions and a larger microbial species pool, diversity ceases to be the primary limiting factor; instead, network complexity (β = 0.61) exerts the strongest direct effect on plant metabolism (**Figures 6C, D**). This indicates that under resource-abundant conditions, the intensity of microbial interactions explains host metabolic diversification better than mere taxonomic richness (Yang et al., 2023). In saline deserts (**Figure 6E**), despite the involvement of network complexity in host regulation, its direct effect is constrained by the severe environmental filtering inherent to salt stress (Cornell et al., 2023). While edaphic factors often directly regulate plant metabolism under non-arid conditions (Tariq et al., 2023), our models underscore the critical intermediary function of the rhizosphere microbiome in desert ecosystems. When baseline resources are severely limited, microbes become the primary conduit linking soil environmental signals to host metabolic regulation.

In stark contrast to alpha-diversity, edaphic factors failed to significantly regulate plant metabolite beta-diversity in any of the three habitats, perfectly mirroring the lack of correlation observed in the linear regression models (**Figures 6A, C, E**). As previously noted, variations in metabolite beta-diversity are partially driven by phenotypic plasticity in response to localized microenvironmental gradients. Although desert ecosystems can be broadly categorized into sandy, saline, and gravelly habitats, profound spatial heterogeneity persists at the micro-scale. In gravelly deserts, localized variations in crevice depth and gravel coverage dictate root-zone hydrothermal dynamics. In saline deserts, minute differences in proximity to salt crusts create stark spatial gradients in root salt exposure. In sandy deserts, microtopographic undulations and the presence of “nurse” shrubs control precipitation redistribution and nutrient islands. Furthermore, local ecological processes—such as neighbor competition, litter accumulation, and biocrust development—exhibit intense spatial patchiness. Plants tailor their metabolic responses to these hyper-local variations, generating discrete metabolic profiles even within a single macro-habitat (Khan, 2025). Concurrently, underlying genetic variations within a population also drive shifts in metabolite beta-diversity (Hayashi et al., 2023). Conspecific individuals often harbor genetic differences in sequences, allele frequencies, and copy numbers associated with secondary metabolic pathways, directly altering the activation thresholds and expression intensities of these networks (Divekar et al., 2022; Marone et al., 2022). The inability of edaphic factors and microbial communities to fully explain metabolite beta-diversity in this study likely stems from the fact that intraspecific metabolic variation possesses a strong, environment-independent genetic component (He et al., 2025), which cannot be resolved by standard environmental metadata. It should be noted that the metabolomic data presented here are correlational and cannot establish causality; direct physiological measurements (e.g., enzyme activities, phytohormones, osmotic adjustment substances) are needed to validate the proposed mechanisms. Future studies must incorporate temporal-dynamic sampling (e.g., across phenological stages) along with highly resolved micro-scale environmental monitoring and population genomics to disentangle the relative contributions of genetic distance, microenvironmental filtering, and time-dependent factors to metabolic phenotypic divergence.

## 5 Conclusion

This study investigated four desert ephemeral species across sandy, saline, and gravelly habitats, elucidating the habitat-specific patterns and differentiation dynamics of plant metabolite composition and rhizosphere microbial interactions. Although lipids and terpenes dominated the metabolomes of all four species, each retained distinct habitat-specific metabolites, predominantly flavonoids. Metabolite alpha-diversity in the Brassicaceae species decreased significantly in the gravelly desert but increased in the saline desert. Conversely, it remained stable in the Boraginaceae species, tentatively suggesting a possible divergence in metabolic plasticity and homeostatic capacity between the two taxonomic families, which warrants further investigation with a larger number of species per family. Regarding rhizosphere microbiomes, the bacterial alpha-diversity associated with Boraginaceae species peaked in the gravelly desert, whereas that of Brassicaceae species peaked in the saline desert. In contrast, rhizosphere fungal alpha-diversity remained independent of host identity, consistently reaching its highest levels in the gravelly desert. Boraginaceae species exhibited maximum microbial network complexity but the lowest proportion of positive correlations in the gravelly desert, reflecting an assembly process driven by resource competition. Brassicaceae species, however, displayed peak network complexity in the saline desert, indicative of stress-induced cooperative interactions. Overall microbial community stability was highest in the gravelly desert across all taxa. Partial least squares path modeling (PLS-PM) confirmed that edaphic factors indirectly regulate plant metabolite alpha-diversity via microbial community diversity, stability, and network complexity. Crucially, microbial diversity emerged as the universal core mediator of plant metabolite alpha-diversity across all three habitats. Conversely, metabolite beta-diversity was not significantly regulated by either edaphic factors or microbial communities in any habitat. Ultimately, this study provides a metabolomics-based perspective on the chemical adaptation strategies of desert ephemeral plants and their complex interaction patterns with rhizosphere microbiomes, providing novel insights into plant-microbe co-adaptation patterns in extreme environments.

## Data Availability

The datasets presented in this study can be found in online repositories. The names of the repository/repositories and accession number(s) can be found below: https://www.ncbi.nlm.nih.gov/, PRJNA1426877.
