# Supplementary material for: Environmental heterogeneity dominates the regulation of metabolites in ephemeral plants by the rhizosphere microbial community
Source: Front Plant Sci. 2026 Jul 27;17:1815311. doi: 10.3389/fpls.2026.1815311 (PMC13454232; doi:10.3389/fpls.2026.1815311)
Supplement: Supplementary file 1 [file DataSheet1.zip › Supplementary files/Supplementary Materials 2.pdf]

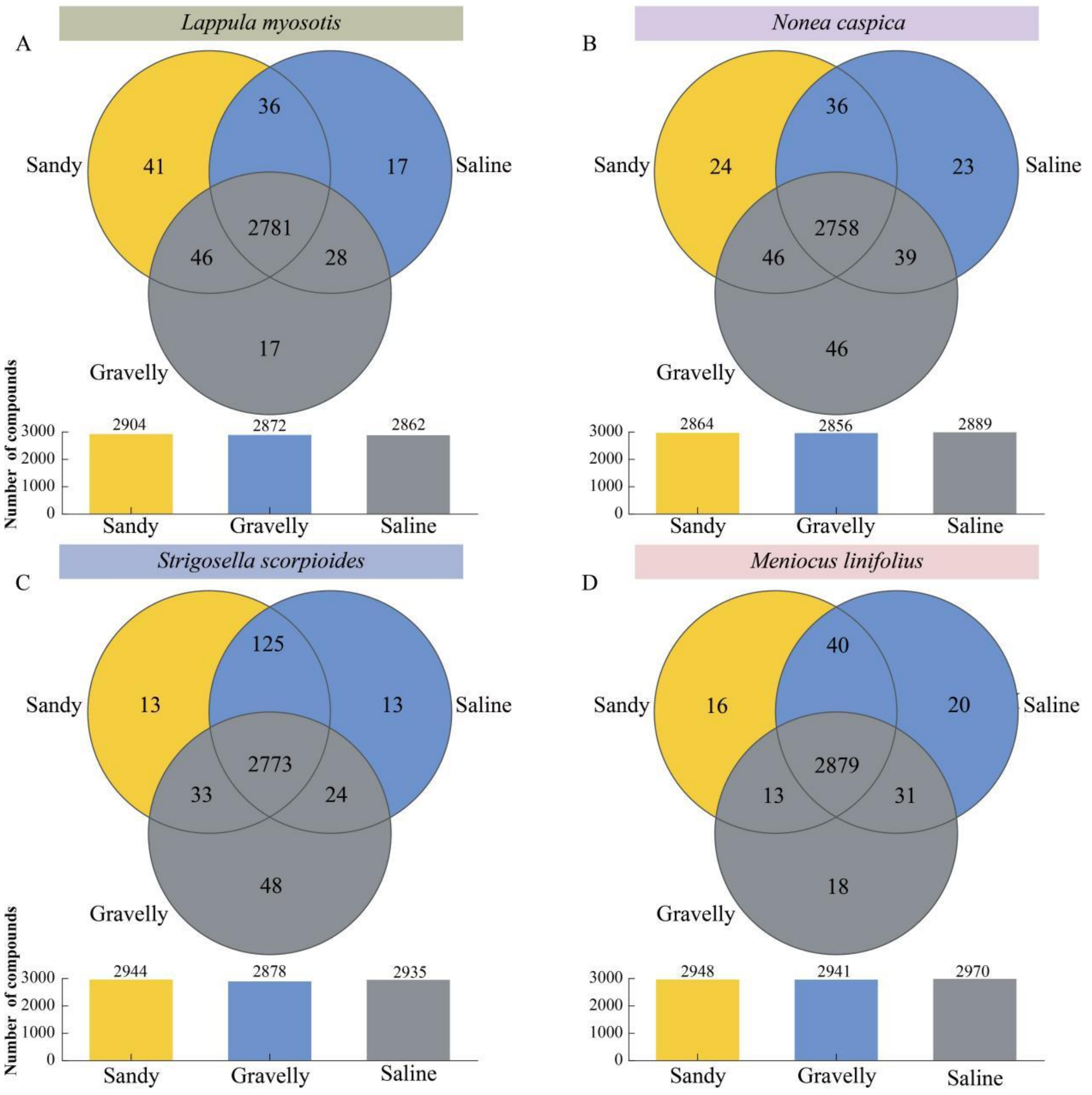

Fig. S1 Statistical analysis of the number of metabolites of *Lappula myosotis*, *Nonea caspica*, *Strigosella scorpioides*, and *Meniocus linifolius* in different habitats. **A:** The number of metabolites of *L. myosotis.* **B:** The number of metabolites of *N. caspica.* **C:** The number of metabolites of *S. scorpioides.* **D:** The number of metabolites of *M. linifolius.*

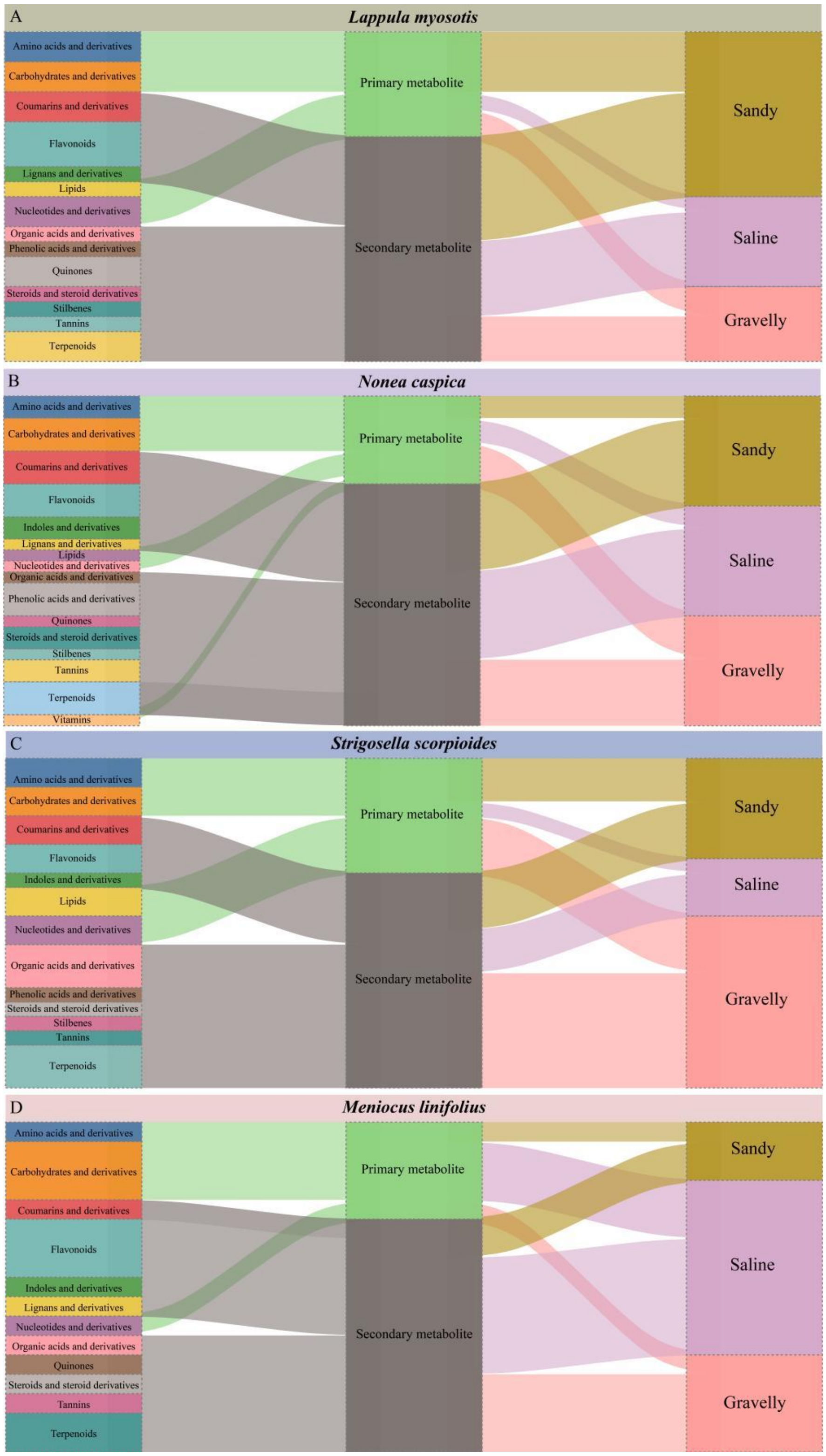

Fig. S2 Statistical classification of the characteristic metabolites of *Lappula myosotis*, *Nonea caspica*, *Strigosella scorpioides*, and *Meniocus linifolius* in different habitats. **A:** Classification of habitat-specific metabolites of *L. myosotis*. **B:** Classification of habitat-specific metabolites of *N. caspica*. **C:** Classification of habitat-specific metabolites of *S. scorpioides*. **D:** Classification of habitat-specific metabolites of *M. linifolius*.

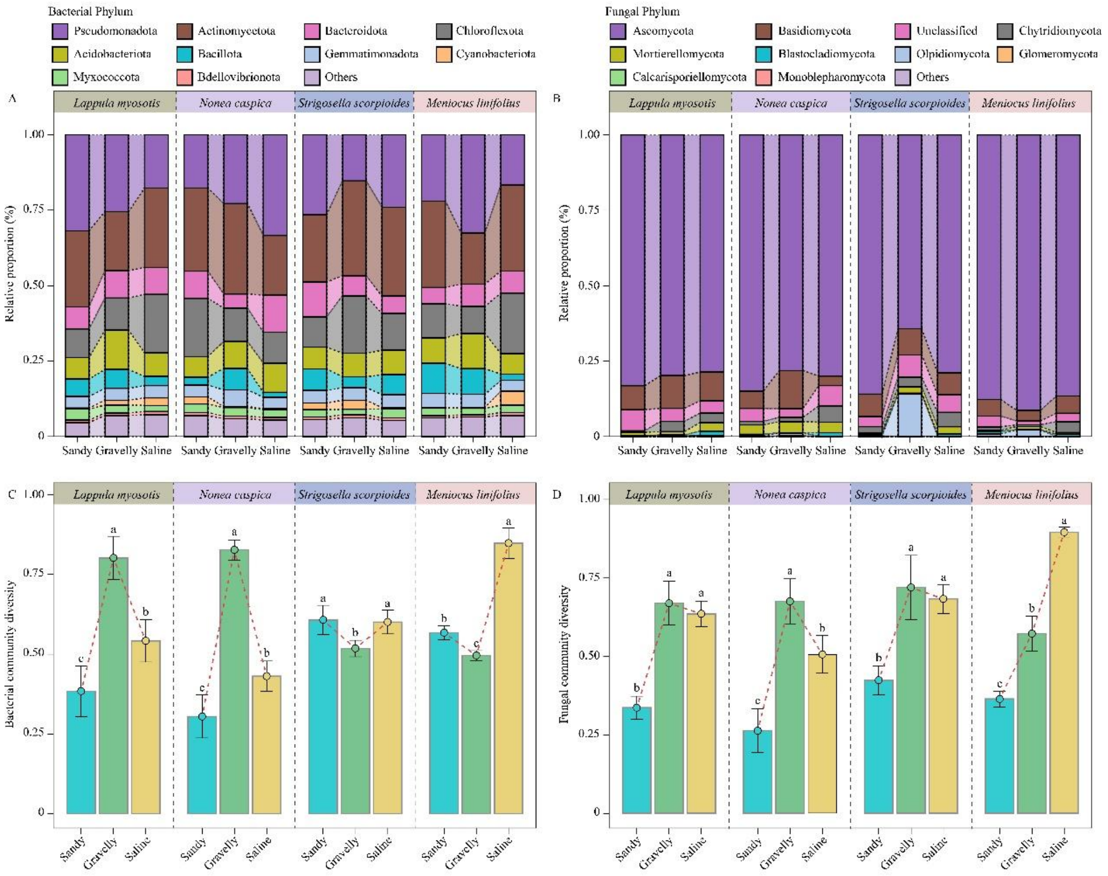

Fig. S3 Composition and diversity of rhizosphere soil microbial communities of *Lappula myosotis, Nonea caspica, Strigosella scorpioides, and Meniocus linifolius* in different habitats. **A:** Rhizosphere bacterial community composition of *L. myosotis*, *N. caspica*, *S. scorpioides*, and *M. linifolius*. B**:** Rhizosphere fungal community composition of *L. myosotis*, *N. caspica*, *S. scorpioides*, and *M. linifolius*. **C:** Rhizosphere bacterial community diversity of *L. myosotis*, *N. caspica*, *S. scorpioides*, and *M. linifolius*. **D:** Rhizosphere fungal community diversity of *L. myosotis*, *N. caspica*, *S. scorpioides*, and *M. linifolius*.

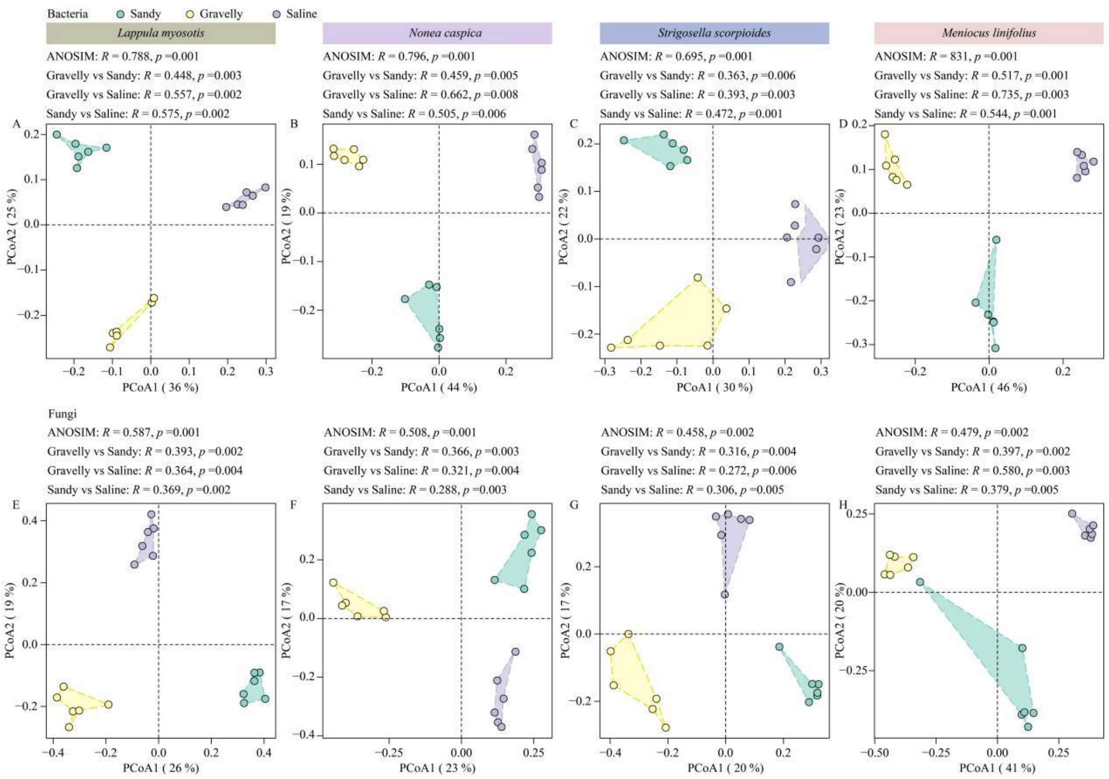

Fig. S4 Principal coordinate analysis (PCoA) of rhizosphere soil microbial communities of four *Lappula myosotis, Nonea caspica, Strigosella scorpioides, and Meniocus linifolius* in different habitats. **A:** Rhizosphere bacterial community of *L. myosotis*. **B:** Rhizosphere bacterial community of *N. caspica*. **C:** Rhizosphere bacterial community of *S. scorpioides*. **D:** Rhizosphere bacterial community of *M. linifolius*. **E:** Rhizosphere fungal community of *L. myosotis*. **F:** Rhizosphere fungal community of *N. caspica*. **G:** Rhizosphere fungal community of *S. scorpioides*. **H:** Rhizosphere fungal community of *M. linifolius*.

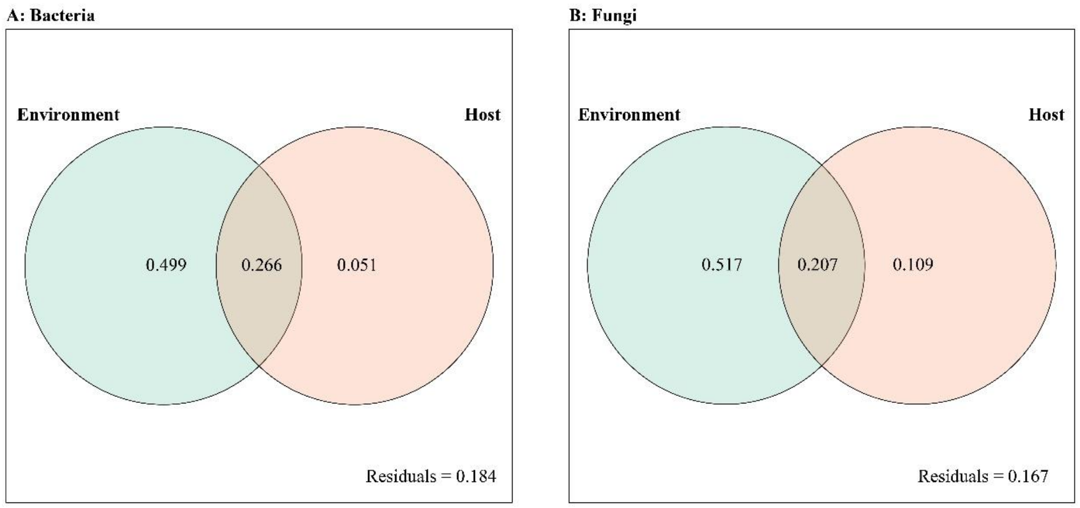

Fig. S5 Variance partitioning analysis (VPA) of rhizosphere soil microbial communities by habitat heterogeneity and host plant identity. **A:** Bacterial community. **B:** Fungal community.

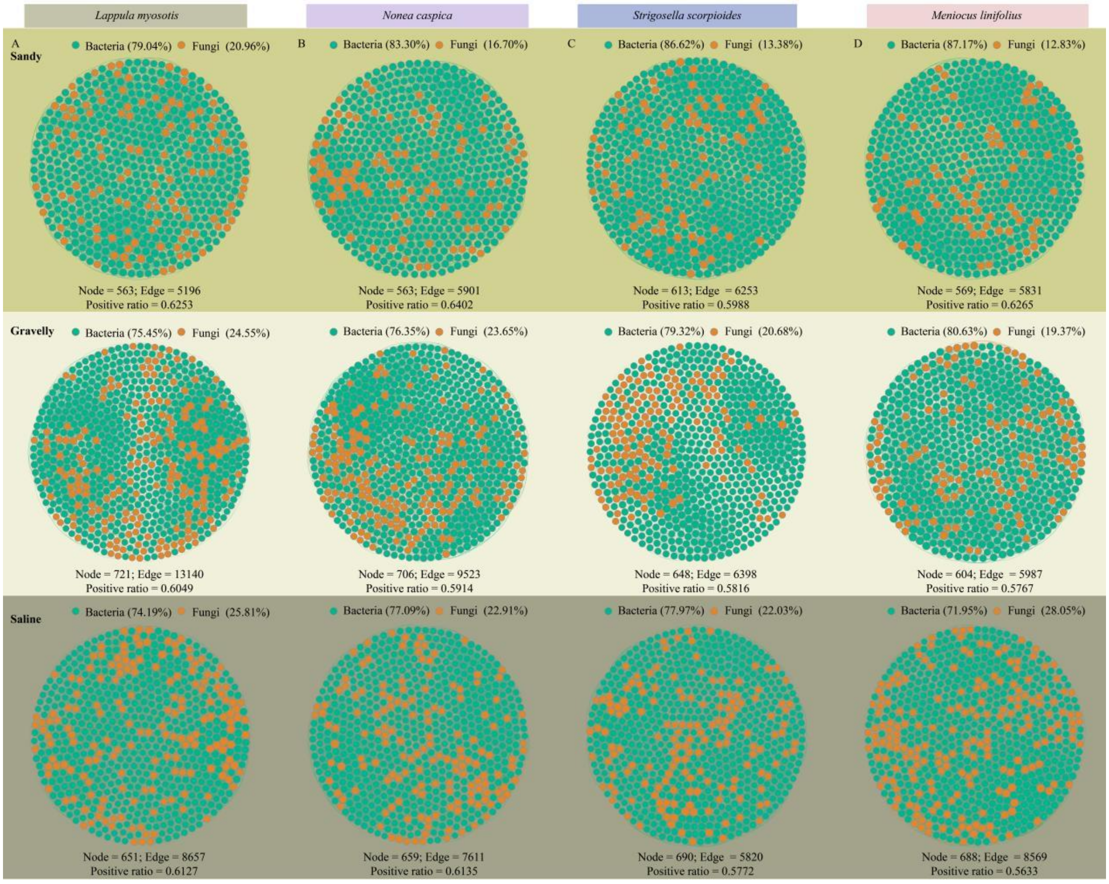

Fig. S6 Relative proportions of bacterial and fungal communities in co-occurrence networks of rhizosphere microbial communities of annual ephemeral plants in sandy desert, saline desert, and gravel desert habitats. **A:** Network structure of rhizosphere soil microbial community of *L. myosotis*. **B:** Network structure of rhizosphere soil microbial community of *N. caspica*. **C:** Network structure of rhizosphere soil microbial community of *S. scorpioides*. **D:** Co-occurrence network of rhizosphere soil microbial community of *M. linifolius*.

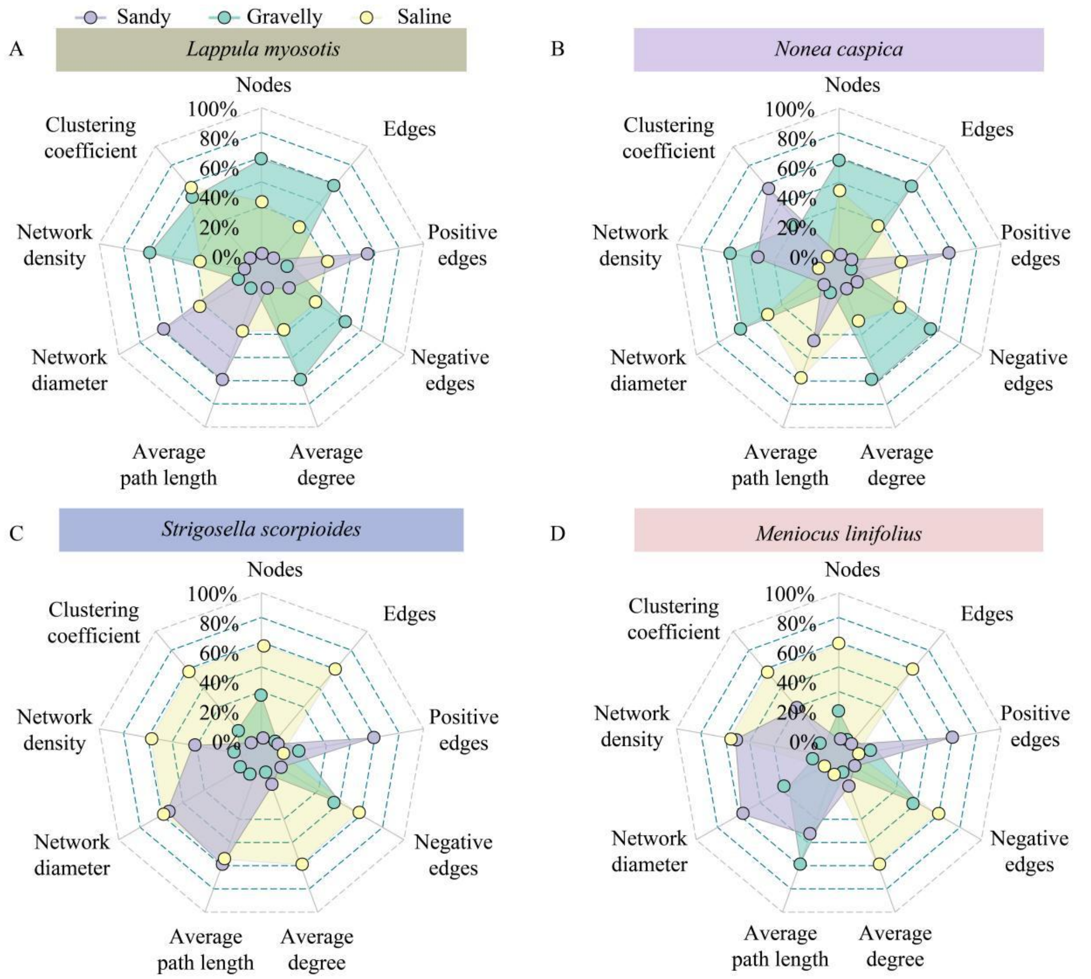

Fig. S7 Topological properties of co-occurrence networks of rhizosphere microbial communities of four ephemeral plants in sandy desert, saline desert, and gravel desert habitats. **A:** Topological properties of the rhizosphere microbial co-occurrence network of *L. myosotis*. **B:** Topological properties of the rhizosphere microbial co-occurrence network of *N. caspica*. **C:** Topological properties of the rhizosphere microbial co-occurrence network of *S. scorpioides*. **D:** Topological properties of the rhizosphere microbial co-occurrence network of *M. linifolius*.

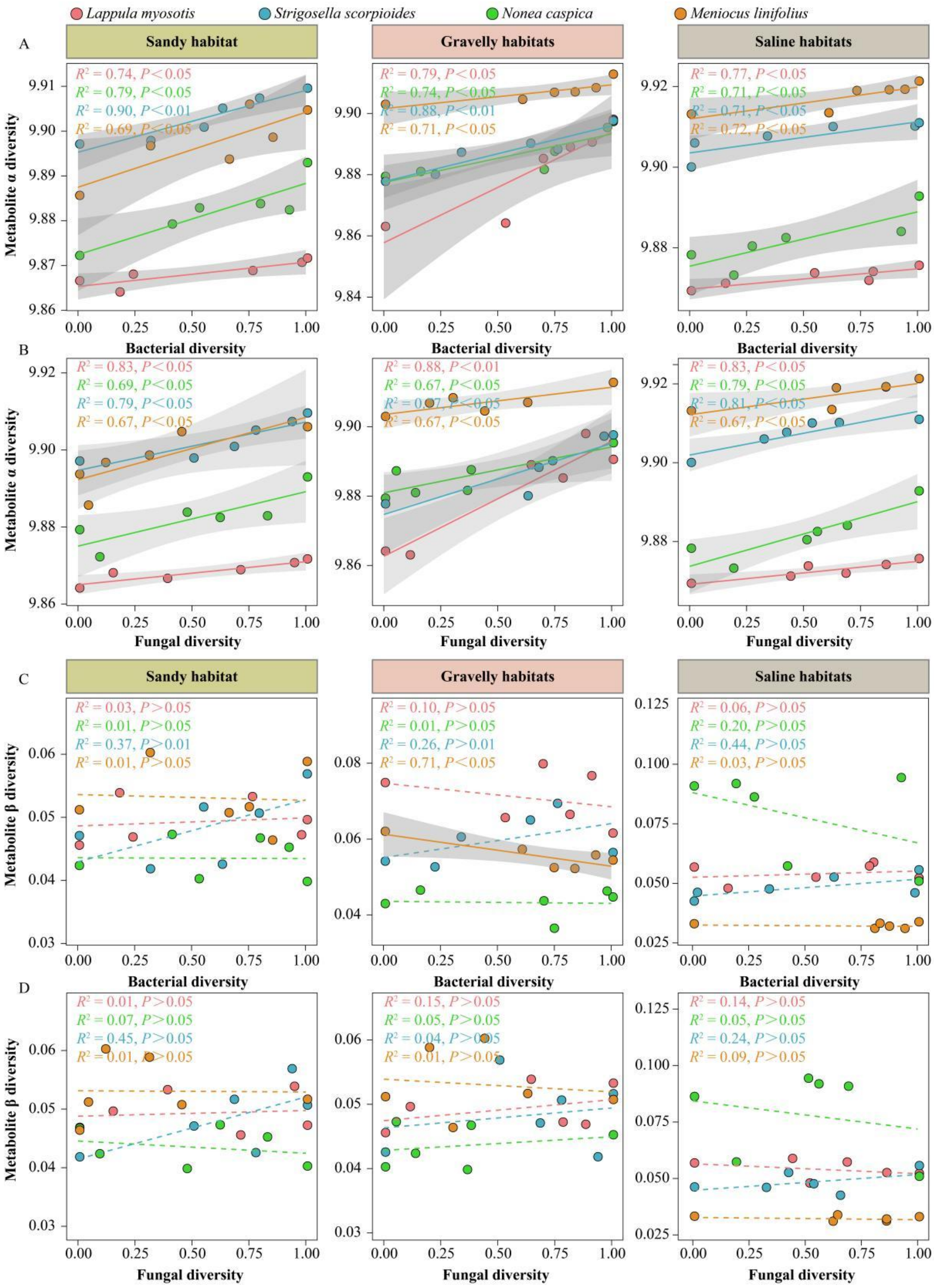

Fig. S8 Correlations between the diversity of rhizosphere microbial communities of four ephemeral plant species and plant metabolites in sandy, saline, and gravel desert habitats. **A:** Correlation between the diversity of rhizosphere soil bacterial communities and the α-diversity of plant metabolites in sandy, saline, and gravel desert habitats. **B:** Correlation between the diversity of rhizosphere soil fungal communities and the α-diversity of plant metabolites in sandy, saline, and gravel desert habitats. **C:** Correlation between the stability of rhizosphere soil bacterial communities and the β-diversity of plant metabolites in sandy, saline, and gravel desert habitats. **D:** Correlation between the diversity of rhizosphere soil fungal communities and the β-diversity of plant metabolites in sandy, saline, and gravel desert habitats.

Table S1. Construct reliability and validity metrics of the PLS-PM measurement models for the three desert habitats.

Habitat	Latent Variable	Indicators	Outer Loading / Weight	Cronbach's α	Composite Reliability (CR)	AVE
Sandy habitat	Soil	SM	0.17	0.68	0.74	0.38
		pH	0.05			
		EC	0.07			
		C	0.47*			
		N	0.52*			
		P	0.56*			
	Diversity	Microbial Shannon	1	1	1	1
	Complexity	Network Complexity	1	1	1	1
	Stability	Network Stability	1	1	1	1
Gravelly habitats	Soil	SM	0.01	0.65	0.72	0.35
		pH	0.03			
		EC	0.27			
		C	0.35			
		N	0.58*			
		P	0.49*			
	Diversity	Microbial Shannon	1	1	1	1
	Complexity	Network Complexity	1	1	1	1
	Stability	Network Stability	1	1	1	1
Saline habitats	Soil	SM	0.09	0.71	0.76	0.4
		pH	0.17			
		EC	0.24			
		C	0.50*			
		N	0.58*			
		P	0.43*			
	Diversity	Microbial Shannon	1	1	1	1
	Complexity	Network Complexity	1	1	1	1
	Stability	Network Stability	1	1	1	1

Note: Latent variables evaluated using a single aggregated/standardized index (Diversity, Complexity, and Stability) have fixed reliability metrics (alpha = 1.00, CR = 1.00, AVE = 1.00). Significance level: *p < 0.05, * *p < 0.01.
